# Modifiable in‐hospital factors for 12‐month global cognition, post‐traumatic stress disorder symptoms, and depression symptoms in adults hospitalized with COVID‐19

**DOI:** 10.1111/irv.13197

**Published:** 2023-09-26

**Authors:** Jin H. Han, James C. Jackson, Onur M. Orun, Samuel M. Brown, Jonathan D. Casey, Lindsay Clark, Sean P. Collins, Kemberlyne Cordero, Adit A. Ginde, Michelle N. Gong, Catherine L. Hough, Theodore J. Iwashyna, Amy L. Kiehl, Alana Lauck, Lindsay M. Leither, Christopher J. Lindsell, Mayur B. Patel, Rameela Raman, Todd W. Rice, Nancy J. Ringwood, Karen L. Sheppard, Matthew W. Semler, B. Taylor Thompson, E. Wesley Ely, Wesley H. Self, for the National Heart, Lung, and Blood Institute PETAL Clinical Trials Network

**Affiliations:** ^1^ Critical Illness, Brain Dysfunction, and Survivorship Center Vanderbilt University Medical Center Nashville Tennessee USA; ^2^ Geriatric Research, Education, and Clinical Center (GRECC) Tennessee Valley Healthcare System Nashville Tennessee USA; ^3^ Department of Emergency Medicine Vanderbilt University Medical Center Nashville Tennessee USA; ^4^ Division of Allergy, Pulmonary, and Critical Care, Department of Medicine Vanderbilt University Medical Center Nashville Tennessee USA; ^5^ Department of Biostatistics Vanderbilt University Medical Center Nashville Tennessee USA; ^6^ Division of Pulmonary/Critical Care Medicine, Department of Medicine Intermountain Medical Center and the University of Utah Salt Lake City Utah USA; ^7^ Division of Geriatrics and Gerontology University of Wisconsin School of Medicine and Public Health Madison Wisconsin USA; ^8^ Geriatric Research, Education, and Clinical Center (GRECC) William S Middleton Memorial Veterans Hospital Madison Wisconsin USA; ^9^ Department of Emergency Medicine University of Colorado School of Medicine Aurora Colorado USA; ^10^ Division of Critical Care, Division of Pulmonary Medicine, Department of Medicine Albert Einstein College of Medicine Bronx New York USA; ^11^ Division of Pulmonary, Allergy, and Critical Care Medicine, Department of Medicine Oregon Health & Science University Portland Oregon USA; ^12^ Division of Pulmonary and Critical Care, Department of Medicine Johns Hopkins University Baltimore Maryland USA; ^13^ Health Policy & Management in the Bloomberg School of Public Health Johns Hopkins University Baltimore Maryland USA; ^14^ Division of Acute Care Surgery, Department of Surgery, Section of Surgical Sciences Vanderbilt University Medical Center Nashville Tennessee USA; ^15^ Vanderbilt Institute for Clinical and Translational Research (VICTR) Vanderbilt University Medical Center Nashville Tennessee USA; ^16^ Division of Pulmonary and Critical Care Medicine Massachusetts General Hospital Boston Massachusetts USA

**Keywords:** depression, long‐COVID, long‐term cognitive impairment, modifiable risk factors, post‐traumatic stress disorder

## Abstract

**Background:**

We sought to identify potentially modifiable in‐hospital factors associated with global cognition, post‐traumatic stress disorder (PTSD) symptoms, and depression symptoms at 12 months.

**Methods:**

This was a multi‐center prospective cohort study in adult hospitalized patients with acute COVID‐19. The following in‐hospital factors were assessed: delirium; frequency of in‐person and virtual visits by friends and family; and hydroxychloroquine, corticosteroid, and remdesivir administration. Twelve‐month global cognition was characterized by the MOCA‐Blind. Twelve‐month PTSD and depression were characterized using the PTSD Checklist for the DSM‐V and Hospital Anxiety Depression Scale, respectively.

**Findings:**

Two hundred three patients completed the 12‐month follow‐up assessments. Remdesivir use was associated with significantly higher cognition at 12 months based on the MOCA‐Blind (adjusted odds ratio [aOR] = 1.98, 95% CI: 1.06, 3.70). Delirium was associated with worsening 12‐month PTSD (aOR = 3.44, 95% CI: 1.89, 6.28) and depression (aOR = 2.18, 95% CI: 1.23, 3.84) symptoms. Multiple virtual visits per day during hospitalization was associated with lower 12‐month depression symptoms compared to those with less than daily virtual visits (aOR = 0.40, 95% CI: 0.19, 0.85).

**Conclusion:**

Potentially modifiable factors associated with better long‐term outcomes included remdesivir use (associated with better cognitive function), avoidance of delirium (associated with less PTSD and depression symptoms), and increased virtual interactions with friends and family (associated with less depression symptoms).

## 1 INTRODUCTION

As of July 2022, over 550 million people have been diagnosed with COVID‐19 worldwide.^1^ A substantial proportion of people with acute COVID‐19 will develop post‐acute sequelae of SARS‐CoV2 (PASC) or Long‐COVID,^2^ which is characterized by symptoms that persist for months after the initial infection.^3^, ^4^, ^5^, ^6^, ^7^, ^8^ A meta‐analysis of 43 studies reported that 22% of patients with COVID‐19 across the entire severity of illness spectrum demonstrated impaired global cognition at least 12 weeks after the acute illness.^9^ Another meta‐analysis reported that 19% developed post‐traumatic stress disorder (PTSD) and 21% developed depression.^10^

To our knowledge, few studies have evaluated modifiable risk factors during hospitalization, such as delirium, medications, and the effect of in‐person and virtual visitation with friends and family on the severity of Long‐COVID symptoms. Identifying in‐hospital modifiable risk factors could assist with the development of strategies to prevent or lessen the contribution of neuropsychological dysfunction to Long‐COVID. To address this gap in knowledge, this study sought to identify potentially modifiable risk factors for global cognition, PTSD symptoms, and depression symptoms 12 months after acute COVID‐19 among adults hospitalized from 34 geographically diverse sites in the United States.

## 2 METHODS

### 2.1 Study design and settings

This was an ancillary study to the Outcomes Related to COVID‐19 treated with Hydroxychloroquine among Inpatients with symptomatic Disease (ORCHID) trial.^11^ ORCHID was a blinded, placebo‐controlled randomized clinical trial that evaluated the efficacy of hydroxychloroquine for adults hospitalized with COVID‐19.^11^ A total of 34 US hospitals in the NHLBI Prevention and Early Treatment of Acute Lung Injury (PETAL) Clinical Trial Network enrolled patients in ORCHID between April 2, 2020, and June 19, 2020. Enrollment was stopped on June 19, 2020, for futility based on recommendations from the Data and Safety Monitoring Board (DSMB), and the primary results demonstrated no difference between hydroxychloroquine and placebo on clinical status and mortality at 14 and 28 days. This ancillary study was called ORCHID‐Brain Outcomes and Psychological Distress (ORCHID‐BUD) and added 12‐month cognitive and psychological assessments to the ORCHID parent trial.

A central institutional review board at Vanderbilt University Medical Center approved ORCHID and ORCHID‐BUD. Patients or their legally authorized representatives provided informed consent; to mitigate the spread of SARS‐CoV2 and to conserve personal protective equipment, informed consent was primarily obtained using an electronic consent procedure, including electronic consent forms (eConsent) and video conferencing for informed consent discussions.

### 2.2 Patient population

The ORCHID trial enrolled adults (≥18 years old) within 48 h of hospitalization with laboratory‐confirmed SARS‐CoV‐2 infection and symptoms of respiratory illness for ≤10 days. Patients with the full spectrum of in‐hospital COVID‐19 severity were enrolled, including those receiving no supplemental oxygen, standard flow oxygen, high flow oxygen, noninvasive ventilation, and invasive mechanical ventilation. Patients from the ORCHID trial who survived to the 28‐day follow‐up and agreed to the follow‐up procedures were included. Patients were excluded from ORCHID‐BUD if they did not speak English or Spanish, were unable to follow simple commands prior to the hospitalization, were non‐verbal, were deaf, were cognitively incapable, or had no contact information available.

### 2.3 Method of measurement

Approximately 1 month after enrollment into the ORCHID trial, research staff conducted phone interviews with patients and/or their family members to obtain baseline data and information about the hospitalization. A past history of dementia, cholinesterase inhibitor medication use (indicating a likely history of dementia), PTSD, and depression prior to the COVID‐19 illness were collected. Pre‐illness (1 month before the COVID‐19 illness) cognition was also characterized using the short form Informant Questionnaire on Cognitive Decline in the Elderly score (IQCODE), which is a 16‐item questionnaire that ranges from 1 (much improved cognition) to 5 (much worse cognition).^12^ Patients were considered to have pre‐illness dementia if the patient had a past history of dementia or cholinesterase use, or an IQCODE score > 3.3.^13^

To characterize social isolation during hospitalization for acute COVID‐19, patients and their families were asked to recall how frequently in‐person visitation by friends or family occurred during hospitalization: once or less than once per week, two to three times per week, four to five times per week, daily, or multiple times per day. Similarly, they were asked to recall how frequently the patient virtually communicated (phone, text messaging, or videoconferencing) using the same scale. They were also asked about the presence of confusion or hallucinations during hospitalization as a marker of delirium.^14^

Estimated intelligence was characterized using the Barona Index.^15^ Severity of illness at enrollment was characterized using the Sequential Organ Failure Assessment.^16^ ICU length of stay, duration of invasive mechanical ventilation, and duration of vasopressor use were collected. Randomization to hydroxychloroquine in the parent ORCHID trial and administration of remdesivir and corticosteroids during hospitalization were also collected.

### 2.4 Outcomes

Experienced neuropsychological raters who were fluent in English and Spanish contacted patients by phone at 12 months. Global cognition at 12 months was characterized using two approaches: a neuropsychological battery and the MOCA‐Blind instrument. The neuropsychological battery consisted of the Wechsler Adult Intelligence Scale‐IV (WAIS‐IV) digit span (attention), Hayling Sentence Completion Task (executive function), Controlled Oral Word Association (verbal fluency), Craft Story – Immediate and Delayed (episodic memory), and WAIS‐IV similarities (abstraction). Cognitive domain scores were normalized for age, sex, education, and/or race. To normalize each cognitive domain score, Z‐scores were calculated as follows:

$$\text{Cognitive domain}\;Z‐\text{score}=\frac{\text{Cognitive domain test score}–\text{Population mean}}{\text{Population standard deviation}}$$

A Z‐score of 1.0 reflects a difference of one standard deviation (SD) from the population mean. Cognitive impairment based on the neuropsychological battery was defined as having two test scores with a Z‐score ≤ −1.5 or one test score with a Z‐score ≤ −2.0 (two test scores ≤ 1.5 SD or one test score ≤ 2.0 SD below the population mean).^17^ Global cognition was quantified by creating a Cognitive Composite Score, which was the mean of the cognitive domain Z‐score.^18^, ^19^ If cognitive domain Z‐scores were missing, then the Cognitive Composite Score was calculated using the non‐missing scores. Global cognition at 12 months was also evaluated with the MOCA‐Blind, which is a brief assessment of global cognition that ranges from 0 to 22; a score less than 18 is commonly used to indicate the presence of cognitive impairment.^20^

PTSD symptoms were characterized using the PTSD Checklist for the DSM‐5 (PCL‐5), a 20‐question questionnaire that asks about symptoms related to PTSD.^21^ The score ranges from 0 to 80, and a score ≥31 is generally considered indicative of PTSD. Depression symptoms were assessed using the Hospital Anxiety and Depression Scale (HADS), with a score ≥8 on the depression subscale indicating depression.^22^ For Spanish speakers, we used the Spanish versions of the neuropsychological tests, MOCA‐Blind, PCL‐5, and HADS.

### 2.5 Data analysis

The parent ORCHID trial included 479 patients and demonstrated no significant effect of hydroxychloroquine on measured clinical outcomes.^11^ Hence, for this ancillary long‐term outcome analysis (ORCHID BUD), the hydroxychloroquine and placebo arms of the ORCHID trial were pooled for analysis. Measures of central tendency and dispersion were reported as medians and interquartile ranges (IQR). Frequency and proportions were reported for categorical variables. The proportion of patients with long‐term cognitive impairment, PTSD, and depression was reported for the entire cohort and in a subset of patients without those pre‐existing conditions.

To evaluate associations between potentially modifiable factors during the acute COVID‐19 hospitalization (delirium, in‐person and virtual visits by family or friends, and medications) and 12‐month global cognition (Cognitive Composite Score and MOCA‐Blind), PTSD symptoms (PCL‐5), and depression symptoms (HADS–Depression subscale), four separate proportional odds models were constructed. The Cognitive Composite Score (based on the neuropsychological testing) and MOCA‐Blind models were adjusted for the following non‐modifiable risk factors: baseline cognition (pre‐illness IQCODE), age at enrollment, non‐Hispanic black race, Hispanic ethnicity, sex, ICU length of stay, estimated intelligence (Barona), and severity of illness at enrollment (SOFA). Duration of mechanical ventilation and vasopressor use were not incorporated in the models because they were highly correlated with ICU length of stay. The models for PCL‐5 score (PTSD) and the HADS–Depression subscale score (depression) also included the aforementioned covariates, but past history of PTSD or depression was incorporated into their respective models instead of the pre‐illness IQCODE. Multiple imputation was used for missing covariates but not for missing outcomes. Delirium, as reported by the patient or their family, was analyzed as a binary variable (present at any time during acute hospitalization versus not present). Receipt of remdesivir, corticosteroids, and hydroxychloroquine were categorized as binary variables (any dose of medication received yes versus no). Due to their distributions, in‐person visits were categorized as yes versus no, and virtual visits were categorized as greater than once daily, daily, and less than daily virtual visits (reference). Proportional odds assumptions were checked graphically. Adjusted odds ratios (aOR) and their 95% confidence intervals (95% CI) were reported. All statistical analyses were conducted with R statistical software, version 4.05 (http://www.r-project.org/).

## 3 RESULTS

Of the 479 patients enrolled in the ORCHID parent study, 392 survived at 12 months and were available for long‐term assessments. An additional 20 patients did not have valid contact information, 91 were lost to follow‐up, 43 refused to participate, 21 did not speak English or Spanish, 9 were excluded due to being incapable of participating in the neurocognitive assessments and surveys, and 5 were excluded for other reasons (Figure S1). A total of 203 patients contributed to this analysis (Table 1). The median (IQR) age was 55 (43.5, 63) years, 94 (46.3%) were female, 84 (41.4%) were Hispanic/Latinx, 47 (23.2%) were non‐Hispanic Black race, 21 (10.3%) met criteria for pre‐existing dementia, 12 (5.9%) had a past history of PTSD, and 36 (17.8%) had a past history of depression. Patient characteristics for those who completed the 12‐month follow‐up compared with those who had no contact information were lost to follow‐up, refused to participate, were non‐English and non‐Spanish speaking, and enrolled in the parent ORCHID trial can be seen in Tables S1 and S2.

**TABLE 1:** Patient characteristics.

Variable	Completed 12‐month follow‐up, *n* = 203
Median (IQR) age, years	55 (43.5, 63.0)
Female Sex	94 (46.3%)
Race/Ethnicity Combined	
Hispanic/Latinx	84 (41.4%)
Non‐Hispanic White	63 (31.0%)
Non‐Hispanic Black	47 (23.2%)
Non‐Hispanic Asian	3 (1.5%)
Non‐Hispanic American Indian or Alaskan Native	3 (1.5%)
Non‐Hispanic Native Hawaiian or Other Pacific Islander	1 (0.5%)
Non‐Hispanic Multiple Race	2 (1.0%)
Median (IQR) education, years	12 (10, 14)
Median (IQR) Barona index (full scale IQ)	98.5 (89.6, 107.6)
Homeless or living only in temporary residences before hospitalization	1 (0.5%)
Median (IQR) BMI, kg/m^2^	32.0 (27.9, 37.2)
Past medical history	
Hypertension	99 (48.8%)
Diabetes mellitus	63 (31.0%)
Coronary artery disease	9 (4.4%)
Chronic obstructive pulmonary disease	13 (6.4%)
Chronic kidney disease	16 (7.9%)
Median (IQR) pre‐illness IQCODE	3.00 (3.00, 3.06)
Past psychiatric history	36 (17.8%)
Dementia^a^	21 (10.3%)
Post‐traumatic stress disorder	12 (5.9%)
Depression	36 (17.8%)
SOFA score at enrollment	2 (1, 3)
Ever in the ICU	63 (31.0%)
Median (IQR) ICU length of stay for all patients, days^b^	0.00 (0.00, 3.50)
Median (IQR) ICU length of stay among those in the ICU, days^b^	10.00 (6.00, 19.50)
Ever mechanically ventilated	34 (16.7%)
Median (IQR) duration of mechanical ventilation among those who were mechanically ventilated, days^b^	12.00 (8.25, 22.00)
Ever vasopressor use	27 (13.3%)
Median (IQR) duration of vasopressor use among those who received vasopressors, days^b^	7.00 (4.50, 18.50)

Abbreviations: IQR, interquartile range; IQ, intelligence quotient as estimated by the Barona Index; IQCODE, Informant Questionnaire on Cognitive Decline in the Elderly; SOFA, Sequential Organ Failure Assessment.

^a^ Pre‐existing dementia was determined by the past history of dementia, home cholinesterase inhibitor use, or an IQCODE > 3.3.

^b^ Length of stays and durations calculated from randomization to 28 days.

**FIGURE 1:**
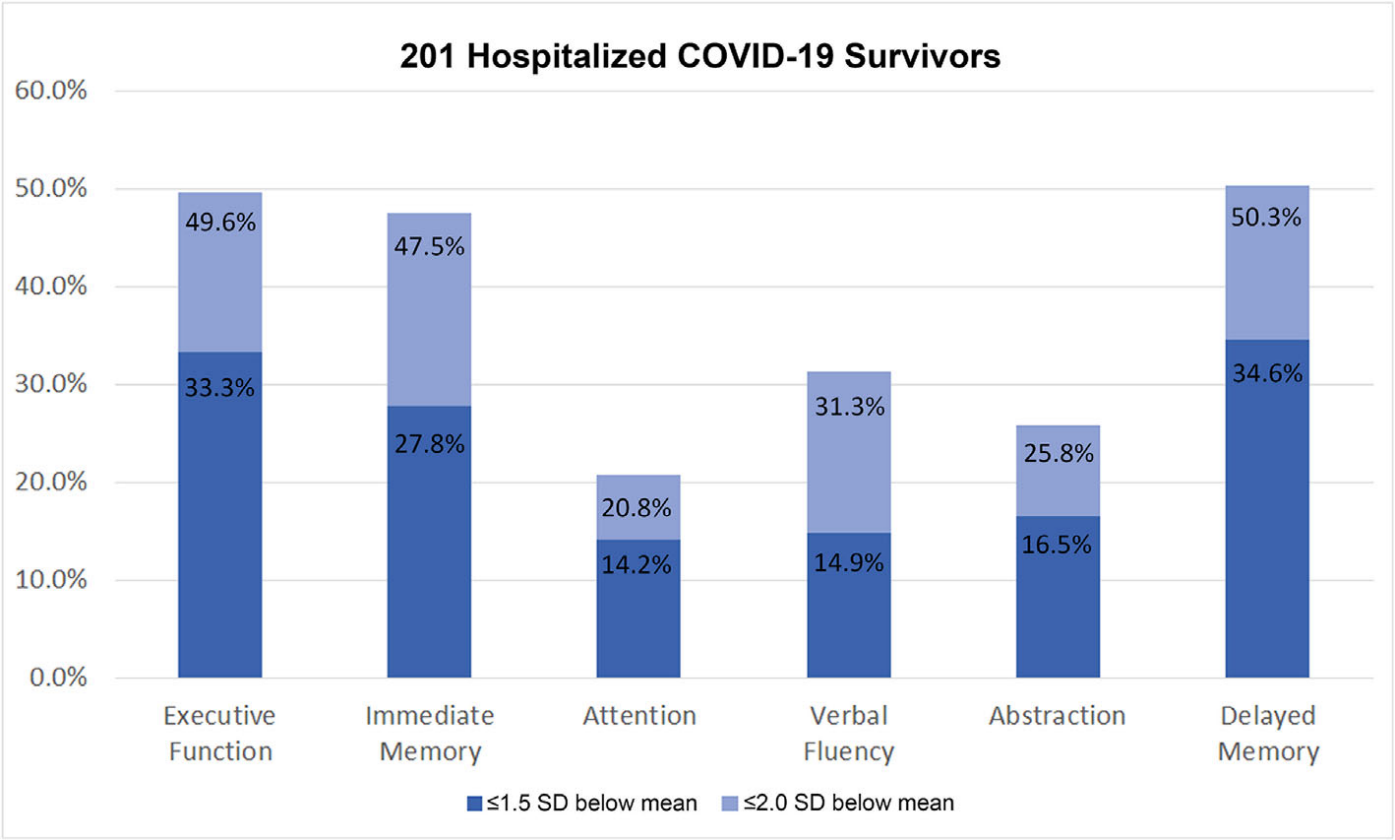
Impairments of individual cognitive domains among 201 patients hospitalized for COVID‐19 who completed neurocognitive testing. Proportions of patients with Z‐scores ≤−1.5 and ≤−2.0, which present 1.5 (moderate impairment) and 2.0 (severe impairment) standard deviations (SD) below the population mean for each cognitive test in all patients. Z‐scores were calculated by subtracting the cognitive domain score from the cognitive test's population mean and dividing by the cognitive test's population standard deviation. Moderate impairments (≤−1.5 SD below the population mean) in 12‐month executive function, immediate memory, and delayed memory were observed in 49.6%, 47.5%, and 50.3% of hospitalized COVID‐19 survivors, respectively. Severe impairments (≤−2.0 SD below the population mean) in 12‐month executive function, immediate memory, and delayed memory were observed in 33.3%, 27.8%, and 34.6% of hospitalized COVID‐19 survivors, respectively.

The 12‐month global cognition and psychological assessment scores and outcomes can be seen in Table 2. A total of 201 patients completed the neuropsychological battery at 12 months with 189 completing all six cognitive domain assessments (Table S3). At 12 months, 137/201 (68.2%) patients met criteria for cognitive impairment based on the Cognitive Composite Score. The cognitive domains predominantly affected were executive function, immediate memory, and delayed memory (Figure 1). Additionally, 134/202 (66.3%) met criteria for cognitive impairment based on the MOCA‐Blind, 32/194 (16.5%) met criteria for PTSD, and 65/194 (33.5%) met criteria for depression. Among those without pre‐existing dementia, PTSD, or depression, 126/181 (69.6%) had new‐onset cognitive impairment as determined by the Cognitive Composite Score, 28/182 (15.4%) had new‐onset PTSD, and 44/159 (27.7%) had new‐onset depression at 12 months, respectively.

**TABLE 2:** Cognition, post‐traumatic stress disorder (PTSD), and depression at 12 months.

Outcomes at 12 months	Total n	Median (IQR) or *n* (%)
Cognitive outcomes		
Cognitive Composite Score, median (IQR)	201	−1.06 (−1.75, −0.54)
Cognitive impairment, *n* (%)	201	137 (68.2%)
New‐onset cognitive impairment, *n* (%)	181	126 (69.6%)
MOCA‐Blind score, median (IQR)	202	16 (13, 18)
MOCA‐Blind < 18 (education adjusted), *n* (%)	202	134 (66.3%)
Individual cognitive domain Z‐scores		
Executive function – Hayling Sentence Completion, median (IQR)	198	−1.31 (−2.56, −0.69)
Immediate memory – Craft Story, median (IQR)	198	−1.33 (−2.18, −0.49)
Attention – WAIS‐IV Digit Span, median (IQR)	197	−0.67 (−1.33, 0.33)
Verbal fluency – COWA, median (IQR)	195	−0.90 (−1.60, −0.10)
Abstraction – WAIS‐IV Similarities, median (IQR)	194	−0.67 (−1.67, 0.00)
Delayed memory – Craft Story, median (IQR)	191	−1.51 (−2.36, −0.64)
Psychological outcomes		
PCL‐5 score, median (IQR)	194	10.0 (4.0, 22.8)
PTSD, *n* (%)	194	32 (16.5%)
New‐onset PTSD, *n* (%)	182	28 (15.4%)
HADS–Depression score, median (IQR)	194	5 (1.3, 9.0)
Depression, *n* (%)	194	65 (33.5%)
New‐onset depression, *n* (%)	159	44 (27.7%)

*Note*: Global cognition was characterized by the Cognitive Composite Score, which took the average of all the cognitive domain Z‐scores and the MOCA‐Blind. Lower scores represent lower cognitive performance. Post‐traumatic stress disorder (PTSD) symptoms were assessed for using the PTSD Checklist for the DSM‐V (PCL‐5), and depression symptoms were assessed for using the Hospital Anxiety Depression Scale (HADS)–Depression Subscale, respectively. Higher scores represented more severe symptoms. New‐onset cognitive impairment, PTSD, and depression were conducted in a subset of patients without pre‐illness dementia, or a past history of PTSD or depression, respectively.

Abbreviations: COWA, Controlled Oral Word Association Test; HADS, Hospital Anxiety and Depression Questionnaire; IQR, interquartile range; WAIS‐IV, Wechsler Adult Intelligence Scale‐IV.

**FIGURE 2:**
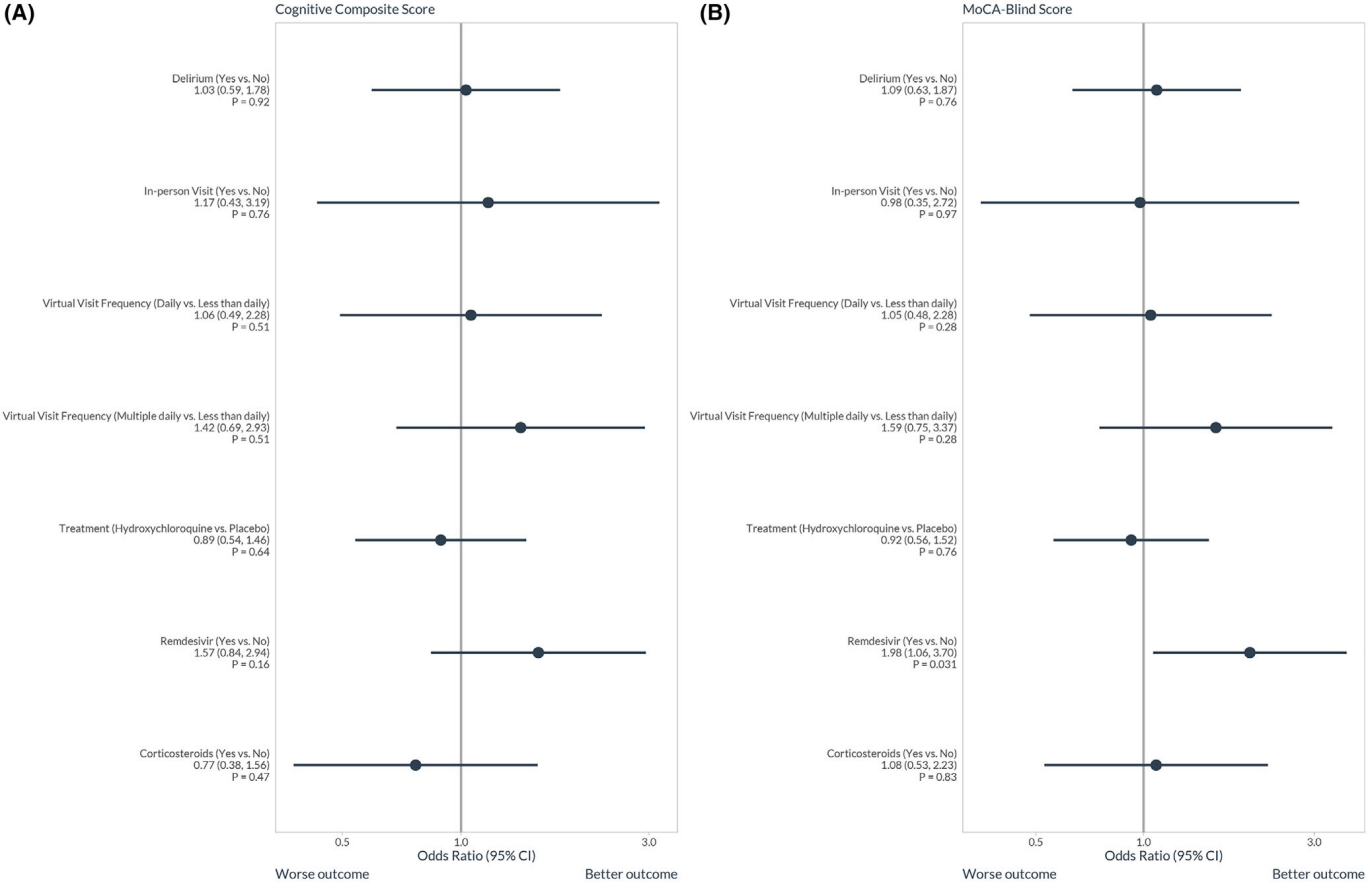
The association between potentially modifiable in‐hospital factors during the treatment of acute COVID‐19 and 12‐month global cognition as characterized by the (A) Cognitive Composite Score based on a detailed neuropsychological battery and (B) MOCA‐Blind in using proportional odds logistic regression. The full models can be seen in Figure S2. Remdesivir use during hospitalization was associated with improved 12‐month global cognition characterized by the MOCA‐Blind (aOR = 1.98, 95% CI: 1.06, 3.70). The aOR for remdesivir use on 12‐month global cognition as characterized by the Cognitive Composite Score was 1.57 (95% CI: 0.84, 2.94).

The frequency and proportion of patients with each potentially modifiable in‐hospital factor can be seen in Table 3. Hydroxychloroquine did not improve any of the 12‐month cognitive or psychological outcomes (Table S4). The multivariable proportion odds logistic regression models for potentially modifiable in‐hospital risk factors on 12‐month global cognition can be seen in Figures 2 and S2. Remdesivir use during hospitalization was associated with significantly higher 12‐month MOCA‐Blind scores (aOR = 1.98, 95% CI: 1.06, 3.70) representing better global cognition, but no significant association was observed for the 12‐month Cognitive Composite Score (aOR = 1.57, 95%: 0.84 to 2.94). There was no association between the 12‐month global cognition (Cognitive Composite Score or MOCA‐Blind) and in‐hospital delirium, in‐person visits, frequency of virtual visits, hydroxychloroquine, or corticosteroids. Modifiable risk factors for the individual cognitive domains can be seen in Figures S3–S8. Remdesivir was associated with significantly higher 12‐month WAIS Similarities scores (aOR = 2.40, 95% CI: 1.23, 4.69) representing better abstract thinking. Corticosteroid use during hospitalization, however, was associated with lower 12‐month WAIS Similarities scores (aOR = 0.42, 95% CI: 0.19, 0.90).

**TABLE 3:** Potentially in‐hospital modifiable factors for 12‐month cognition, post‐traumatic stress disorder, and depression.

Potentially in‐hospital modifiable risk factors	*n* (%)
Delirium during the acute COVID‐19 hospitalization	115 (56.7%)
Frequency of in‐person visits from friends and family during the acute COVID‐19 hospitalization (for all patients)	
Never	185 (91.6%)
Once or less than once per week	3 (1.5%)
2–3 times per week	5 (2.5%)
Daily	9 (4.5%)
Frequency of virtual visits from friends and family during the acute COVID‐19 hospitalization (for all patients)	
Never	7 (3.5%)
Once or less than once per week	7 (3.5%)
2–3 times per week	16 (7.9%)
4–5 times per week	4 (2.0%)
Daily	62 (30.7%)
Multiple times per day	106 (52.5%)
In‐hospital COVID‐19 treatment	
Hydroxychloroquine	97 (47.8%)
Corticosteroids	29 (14.3%)
Remdesivir	45 (22.2%)

**FIGURE 3:**
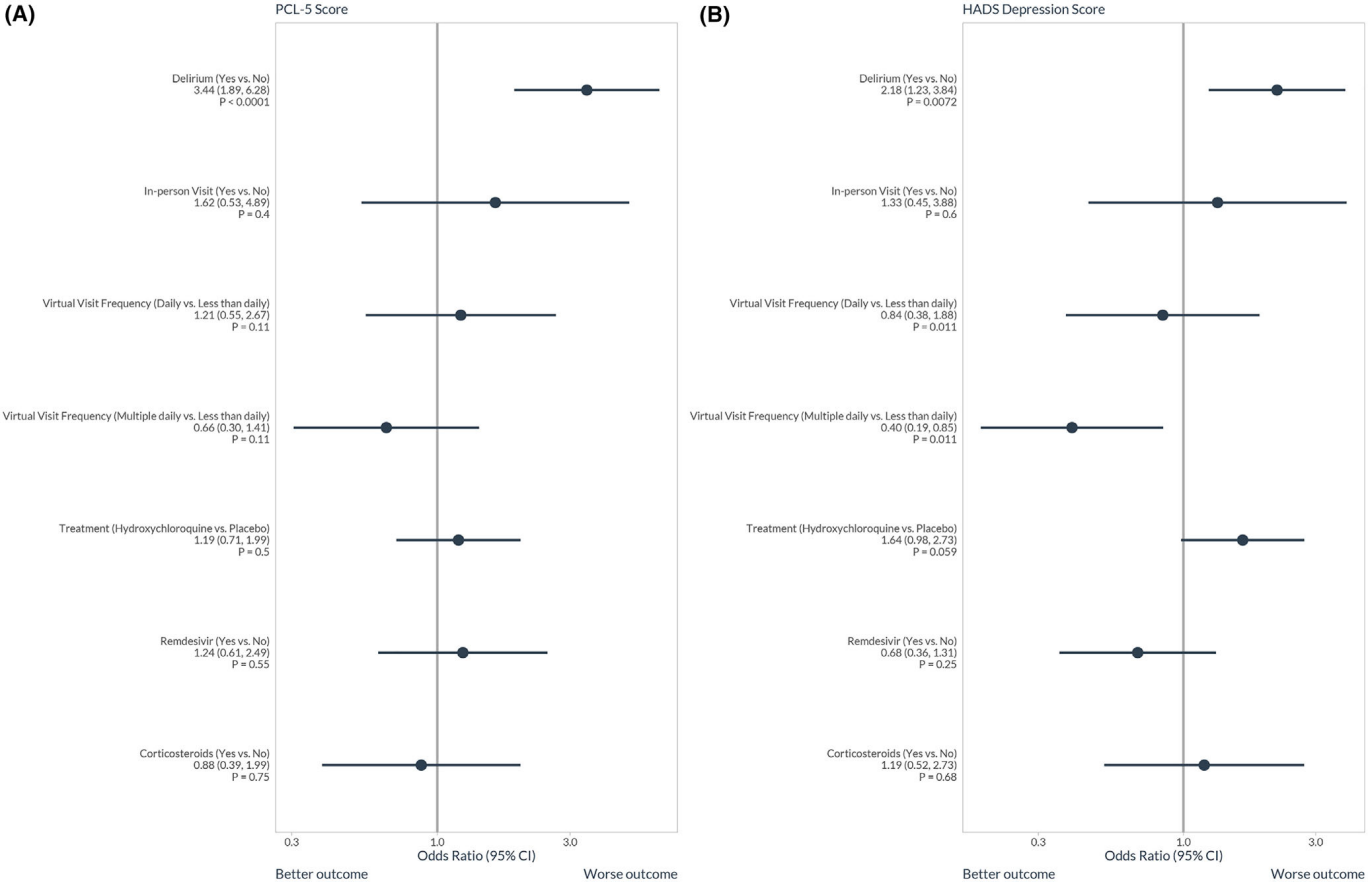
The association between potentially modifiable in‐hospital factors during the treatment of acute COVID‐19 and 12‐month (A) post‐traumatic stress disorder (PTSD) symptoms as characterized by the PTSD Checklist for the DSM‐V (PCL‐5) and (B) depression symptoms as characterized by Hospital Anxiety Depression Scale (HADS)–Depression subscale using proportional odds logistic regression. The full model can be seen in Figure S9. Delirium during hospitalization was associated with higher 12‐month PTSD symptom severity (aOR = 3.44, 95% CI: 1.89, 6.28) and higher depression symptom severity (aOR = 2.18, 95% CI: 1.23, 3.84). Having multiple virtual visits per day from friends or family during hospitalization was associated with better 12‐month depression symptom severity (aOR = 0.40, 95% CI: 0.19, 0.85) compared to those who had less than daily virtual visits.

The multivariable proportion odds logistic regression models for potentially modifiable in‐hospital risk factors on 12‐month PTSD and depression symptoms can be seen in Figures 3 and S9. Self‐ or family‐reported delirium during hospitalization was the only modifiable risk factor associated with higher 12‐month PCL‐5 scores (higher PTSD symptom severity, aOR = 3.44, 95% CI: 1.89, 6.28). Delirium was also associated with higher HADS–Depression subscale scores (worsening depression symptom severity, aOR = 2.18, 95% CI: 1.23, 3.84). Having multiple virtual visits per day from friends or family during hospitalization was associated with lower HADS–Depression subscale score (better depression symptom severity, aOR = 0.40, 95% CI: 0.19, 0.85) compared to those who had less than daily virtual visits. However, having daily virtual visits per day was not significantly associated with the HADS–Depression score (aOR = 0.84, 95% CI: 0.38, 1.88).

## 4 DISCUSSION

The burden of long‐term cognitive impairment, PTSD, and depression at 12 months is high, affecting millions of hospitalized COVID‐19 survivors worldwide. Identifying modifiable risk factors is the critical first step to mitigating these outcomes. These findings may also help inform health policy and responses to future pandemics with novel respiratory viruses. While hydroxychloroquine did not significantly affect the cognitive and psychological mental health outcomes, other potentially modifiable risk factors were identified, such as remdesivir treatment (improved cognition), delirium as reported by the patient or family (worse PTSD and depression symptoms), and multiple virtual visits per day with friends and family (less depression symptoms). Our data may help inform strategies to prevent and treat these negative long‐term outcomes, but additional study is needed, including conducting clinical trials that include PASC or Long‐COVID as long‐term outcomes.

To our knowledge, few studies have evaluated modifiable risk factors on post‐COVID‐19 long‐term cognitive and psychological outcomes. Remdesivir may improve 12‐month cognition as it was associated with higher cognitive performance based on the MOCA‐Blind (global cognition) and WAIS Similarity test (abstraction). Higher frequency of virtual visits by phone, text, or videoconferencing during the hospital course was associated with decreased 12‐month depression symptoms, suggesting that frequent interaction with the outside world during hospitalization may mitigate adverse psychological outcomes associated with COVID‐19. Therefore, every effort should be made to encourage COVID‐19 patients who are hospitalized to virtually interact with friends and families. We also observed that delirium was associated with worse PTSD and depression symptoms at 12 months. Administering non‐pharmacologic multi‐component delirium prevention protocols may help mitigate these adverse psychological outcomes and could be considered.^23^, ^24^ These interventions include performing cognitive stimulating activities, enhancing mobilization, normalizing sleep–wake cycles that are often reversed in delirium, and using glasses and hearing aids to correct sensory impairment as needed.

We observed a high proportion of patients with 12‐month cognitive impairment (68%). A recent meta‐analysis of 43 studies reported a pooled prevalence of 22% of long‐term cognitive impairment in COVID‐19 survivors across all ages in both the outpatient and inpatient settings. The high prevalence of cognitive impairment observed in our cohort could be due to several factors. First, our patient population included patients with a higher severity of acute COVID‐19 than most prior studies, with all patients hospitalized and 31% treated in the ICU. Second, the population was enrolled early in the pandemic, before the availability of COVID‐19 vaccines and largely before any effective acute treatments for COVID‐19 were known; only 14% of patients received corticosteroids and 22% received remdesivir. Thus, this study reported on outcomes for survivors of severe COVID‐19 from the original wild‐type SARS‐CoV‐2 virus that was largely untreated in the acute phase. We also observed that 17% and 34% of hospitalized COVID‐19 survivors met criteria for PTSD and depression at 12 months, respectively. Our findings align with a meta‐analysis of 33 studies that reported a 19% prevalence of PTSD and 21% prevalence of depression after the COVID‐19 illness.^10^

We also examined which individual cognitive domains were particularly affected after COVID‐19 to fully characterize the oft‐reported “brain fog.” We observed prominent impairments in executive function and immediate and delayed memory—domains previously reported to be affected by critical illness and acute respiratory distress syndrome.^25^ These findings are consistent with a recent systematic review, which reported that executive function and episodic memory were the cognitive domains most frequently impaired after acute COVID‐19 infection.^26^ Other studies have also reported deficits in attention,^27^, ^28^, ^29^ processing speed,^27^, ^28^, ^30^ verbal fluency,^27^ and visuospatial.^28^, ^31^ More research is needed to fully understand the types of cognitive impairment patients have after COVID‐19. Such data will facilitate tailored cognitive rehabilitation interventions to help COVID‐19 patients overcome their cognitive deficits.

The strengths of our study included enrollment of patients from 34 geographically diverse sites in the United States, with the majority of participants coming from ethnic or racial minority groups disproportionately affected by COVID‐19. We also performed an extensive phone battery to closely examine individual cognitive domains in both English and Spanish. However, our study had several limitations. First, we only included those who were hospitalized due to acute COVID‐19, and our results cannot be generalized to outpatients with mild‐to‐moderate COVID‐19. Second, our cohort was enrolled early in the pandemic before COVID‐19 vaccines and most treatments were available. Our findings illustrate long‐term outcomes for unvaccinated COVID‐19 patients who mostly did not receive antiviral or immunomodulatory therapy in the acute setting. Third, despite making numerous attempts, our rate of loss to follow‐up was 23%. Of those contacted, 43 (11%) refused to participate. While severity of illness was similar to those with complete follow‐up, patients who were lost‐to‐follow‐up were more likely to be younger, male, and Hispanic/Latinx ethnicity. Fourth, the interpretation of our risk factor analyses may be biased by residual confounding. For example, we did not account for social support, which may have confounded the association between virtual visits and 12‐month depression. Fifth, our assessment of delirium and frequency of in‐person or virtual visitation relied on retrospective reports, which could be affected by recall bias.

In conclusion, we identified potentially modifiable factors during acute COVID‐19 associated with better long‐term outcomes: remdesivir use (associated with better cognitive function), avoidance of delirium (associated with less PTSD and depression symptoms), and increased virtual interactions with friends and family (associated with less depression symptoms). Additional research evaluating strategies to prevent neuropsychological sequelae associated with Long‐COVID should consider antiviral medications, delirium avoidance, and optimizing social interactions for patients early in the illness.

## AUTHOR CONTRIBUTIONS

**Jin H. Han**: Conceptualization; data curation; formal analysis; funding acquisition; investigation; methodology; project administration; supervision; visualization; writing—original draft; writing—review and editing. **James C. Jackson**: Funding acquisition; investigation; methodology; supervision; writing—review and editing. **Onur M. Orun**: Formal analysis; methodology; validation; writing—review and editing. **Samuel M. Brown**: Data curation; writing—review and editing. **Jonathan D. Casey**: Data curation; writing—review and editing. **Lindsay Clark**: Methodology; writing—review and editing. **Sean P. Collins**: Data curation; project administration; writing—review and editing. **Kemberlyne Cordero**: Data curation; writing—review and editing. **Adit A. Ginde**: Data curation; writing—review and editing. **Michelle N. Gong**: Data curation; writing—review and editing. **Catherine L. Hough**: Data curation; writing—review and editing. **Theodore J. Iwashyna**: Writing—review and editing. **Amy L. Kiehl**: Data curation; methodology; project administration; writing—review and editing. **Alana Lauck**: Data curation; project administration; writing—review and editing. **Lindsay M. Leither**: Data curation; writing—review and editing. **Christopher J. Lindsell**: Formal analysis; writing—review and editing. **Mayur B. Patel**: Writing—original draft; writing—review and editing. **Rameela Raman**: Formal analysis; funding acquisition; methodology; validation. **Todd W. Rice**: Data curation; funding acquisition; writing—review and editing. **Nancy J. Ringwood**: Data curation; project administration; writing—review and editing. **Karen L. Sheppard**: Data curation; project administration; writing—review and editing. **Matthew W. Semler**: Data curation; writing—review and editing. B. Taylor Thompson: Data curation; Funding acquisition; project administration; writing—review and editing. **E. Wesley Ely**: Conceptualization; funding acquisition; methodology; writing—review and editing. **Wesley H. Self**: Conceptualization; data curation; funding acquisition; project administration; resources; supervision; writing—original draft; writing—review and editing.

## CONFLICT OF INTEREST STATEMENT

The authors have no conflicts of interest to disclose.

### PEER REVIEW

The peer review history for this article is available at https://www.webofscience.com/api/gateway/wos/peer-review/10.1111/irv.13197.

## Supporting information


**Table S1.** Patient characteristics of those who were and were not enrolled.
**Table S2.** Patient characteristics of those who were enrolled in ORCHID‐BUD and ORCHID.
**Table S3.** The number of cognitive domains tested for each patient.
**Table S4.** Cognitive and psychological outcomes ‐ hydroxychloroquine vs placebo.
**Figure S1.** Enrollment flow diagram.
**Figure S2.** Potentially modifiable in‐hospital factors for 12‐month global cognition.
**Figure S3.** Potentially modifiable in‐hospital factors for 12‐month executive function.
**Figure S4.** Potentially modifiable in‐hospital factors for 12‐month immediate memory.
**Figure S5.** Potentially modifiable in‐hospital factors for 12‐month verbal fluency.
**Figure S6.** Potentially modifiable in‐hospital factors for 12‐month attention.
**Figure S7.** Potentially modifiable in‐hospital factors for 12‐month abstraction.
**Figure S8.** Potentially modifiable in‐hospital factors for 12‐month delayed memory.
**Figure S9.** Potentially modifiable in‐hospital factors for 12‐month post‐traumatic stress disorder and depression.

## Data Availability

The data that support the findings of this study will be openly available at Biolincc 1 year after publication at biolincc.nhlbi.nih.gov/studies/petal_orchid.

## References

[1] Available at: https://gisanddata.maps.arcgis.com/apps/opsdashboard/index.html#/bda7594740fd40299423467b48e9ecf6. May 15, 2022.

[2] Nalbandian A , Sehgal K , Gupta A , et al. Post‐acute COVID‐19 syndrome. Nat Med. 2021;27(4):601‐615. doi:10.1038/s41591-021-01283-z 33753937 PMC8893149

[3] Han JH , Womack KN , Tenforde MW , et al. Associations between persistent symptoms after mild COVID‐19 and long‐term health status, quality of life, and psychological distress. Influenza Other Respi Viruses. 2022;16(4):680‐689. doi:10.1111/irv.12980 PMC9111447 35347854

[4] Logue JK , Franko NM , McCulloch DJ , et al. Sequelae in adults at 6 months after COVID‐19 infection. JAMA Netw Open. 2021;4(2):e210830. doi:10.1001/jamanetworkopen.2021.0830 33606031 PMC7896197

[5] Garrigues E , Janvier P , Kherabi Y , et al. Post‐discharge persistent symptoms and health‐related quality of life after hospitalization for COVID‐19. J Infect. 2020;81(6):e4‐e6. doi:10.1016/j.jinf.2020.08.029 32853602 PMC7445491

[6] Carfi A , Bernabei R , Landi F . Gemelli against C‐P‐ACSG. Persistent symptoms in patients after acute COVID‐19. Jama. 2020;324(6):603‐605. doi:10.1001/jama.2020.12603 32644129 PMC7349096

[7] Huang C , Huang L , Wang Y , et al. 6‐month consequences of COVID‐19 in patients discharged from hospital: a cohort study. Lancet. 2021;397(10270):220‐232. doi:10.1016/S0140-6736(20)32656-8 33428867 PMC7833295

[8] Lopez‐Leon S , Wegman‐Ostrosky T , Perelman C , et al. More than 50 long‐term effects of COVID‐19: a systematic review and meta‐analysis. Sci Rep. 2021;11(1):16144. doi:10.1038/s41598-021-95565-8 34373540 PMC8352980

[9] Ceban F , Ling S , Lui LMW , et al. Fatigue and cognitive impairment in post‐COVID‐19 syndrome: a systematic review and meta‐analysis. Brain Behav Immun. 2022;101:93‐135. doi:10.1016/j.bbi.2021.12.020 34973396 PMC8715665

[10] Marchi M , Grenzi P , Serafini V , et al. Psychiatric symptoms in long‐COVID patients: a systematic review. Front Psych. 2023;14:1138389. doi:10.3389/fpsyt.2023.1138389 PMC10320160 37415689

[11] Self WH , Semler MW , Leither LM , et al. Effect of hydroxychloroquine on clinical status at 14 days in hospitalized patients with COVID‐19: a randomized clinical trial. Jama. 2020;324(21):2165‐2176. doi:10.1001/jama.2020.22240 33165621 PMC7653542

[12] Holsinger T , Deveau J , Boustani M , Williams JW Jr . Does this patient have dementia? Jama. 2007;297(21):2391‐2404. doi:10.1001/jama.297.21.2391 17551132

[13] Jorm AF . The Informant Questionnaire on cognitive decline in the elderly (IQCODE): a review. Int Psychogeriatr/IPA. 2004;16(3):275‐293. doi:10.1017/S1041610204000390 15559753

[14] Sands MB , Dantoc BP , Hartshorn A , Ryan CJ , Lujic S . Single Question in Delirium (SQiD): testing its efficacy against psychiatrist interview, the Confusion Assessment Method and the Memorial Delirium Assessment Scale. Palliat Med. 2010;24(6):561‐565. doi:10.1177/0269216310371556 20837733

[15] Barona A , Reynolds CR , Chastain R . A demographically based index of premorbid intelligence for the WAIS‐R. J Consult Clin Psychol. 1984;35:341‐345.

[16] Vincent JL , de Mendonca A , Cantraine F , et al. Use of the SOFA score to assess the incidence of organ dysfunction/failure in intensive care units: results of a multicenter, prospective study. Working group on “sepsis‐related problems” of the European Society of Intensive Care Medicine. Crit Care Med. 1998;26(11):1793‐1800. doi:10.1097/00003246-199811000-00016 9824069

[17] Christie JD , Biester RC , Taichman DB , et al. Formation and validation of a telephone battery to assess cognitive function in acute respiratory distress syndrome survivors. J Crit Care. 2006;21(2):125‐132. doi:10.1016/j.jcrc.2005.11.004 16769455

[18] Donohue MC , Sperling RA , Salmon DP , et al. The preclinical Alzheimer cognitive composite: measuring amyloid‐related decline. JAMA Neurol. 2014;71(8):961‐970. doi:10.1001/jamaneurol.2014.803 24886908 PMC4439182

[19] Jonaitis EM , Koscik RL , Clark LR , et al. Measuring longitudinal cognition: individual tests versus composites. Alzheimers Dement (Amst). 2019;11(1):74‐84. doi:10.1016/j.dadm.2018.11.006 31673596 PMC6816509

[20] Pendlebury ST , Welch SJ , Cuthbertson FC , Mariz J , Mehta Z , Rothwell PM . Telephone assessment of cognition after transient ischemic attack and stroke: modified telephone interview of cognitive status and telephone Montreal Cognitive Assessment versus face‐to‐face Montreal Cognitive Assessment and neuropsychological battery. Stroke J Cereb Circ. 2013;44(1):227‐229. doi:10.1161/STROKEAHA.112.673384 PMC5593099 23138443

[21] Weathers FW , Litz BT , Keane TM , Palmieri PA , Marx BP , Schnurr PP . The PTSD Checklist for DSM‐5 (PCL‐5). Available at: www.ptsd.va.gov

[22] Zigmond AS , Snaith RP . The hospital anxiety and depression scale. Acta Psychiatr Scand. 1983;67(6):361‐370. doi:10.1111/j.1600-0447.1983.tb09716.x 6880820

[23] Inouye SK , Bogardus ST Jr , Charpentier PA , et al. A multicomponent intervention to prevent delirium in hospitalized older patients. N Engl J Med. 1999;340(9):669‐676. doi:10.1056/NEJM199903043400901 10053175

[24] Hshieh TT , Yue J , Oh E , et al. Effectiveness of multicomponent nonpharmacological delirium interventions: a meta‐analysis. JAMA Intern Med. 2015;175(4):512‐520. doi:10.1001/jamainternmed.2014.7779 25643002 PMC4388802

[25] Hopkins RO , Weaver LK , Pope D , Orme JF , Bigler ED , Larson LV . Neuropsychological sequelae and impaired health status in survivors of severe acute respiratory distress syndrome. Am J Respir Crit Care Med. 1999;160(1):50‐56. doi:10.1164/ajrccm.160.1.9708059 10390379

[26] Tavares‐Junior JWL , de Souza ACC , Borges JWP , et al. COVID‐19 associated cognitive impairment: a systematic review. Cortex. 2022;152:77‐97. doi:10.1016/j.cortex.2022.04.006 35537236 PMC9014565

[27] Vannorsdall TD , Brigham E , Fawzy A , et al. Cognitive dysfunction, psychiatric distress, and functional decline after COVID‐19. J Acad Consult Liaison Psychiatr. 2022;63(2):133‐143. doi:10.1016/j.jaclp.2021.10.006 PMC8591857 34793996

[28] Delgado‐Alonso C , Valles‐Salgado M , Delgado‐Alvarez A , et al. Cognitive dysfunction associated with COVID‐19: a comprehensive neuropsychological study. J Psychiatr Res. 2022;150:40‐46. doi:10.1016/j.jpsychires.2022.03.033 35349797 PMC8943429

[29] Damiano RF , Caruso MJG , Cincoto AV , et al. Post‐COVID‐19 psychiatric and cognitive morbidity: preliminary findings from a Brazilian cohort study. Gen Hosp Psychiatry. 2022;75:38‐45. doi:10.1016/j.genhosppsych.2022.01.002 35134702 PMC8734055

[30] Ferrucci R , Dini M , Rosci C , et al. One‐year cognitive follow‐up of COVID‐19 hospitalized patients. Eur J Neurol. 2022;29(7):2006‐2014. doi:10.1111/ene.15324 35285122 PMC9111730

[31] Mendez R , Balanza‐Martinez V , Luperdi SC , et al. Short‐term neuropsychiatric outcomes and quality of life in COVID‐19 survivors. J Intern Med. 2021;290(3):621‐631. doi:10.1111/joim.13262 33533521 PMC8013333

