# Supplementary material for: Modifiable in‐hospital factors for 12‐month global cognition, post‐traumatic stress disorder symptoms, and depression symptoms in adults hospitalized with COVID‐19
Source: Influenza Other Respir Viruses. 2023 Sep 26;17(9):e13197. doi: 10.1111/irv.13197 (PMC10522479; doi:10.1111/irv.13197)
Supplement: Supplementary file 1 — Table S1. Patient characteristics of those who were and were not enrolled. Table S2. Patient characteristics of those who were enrolled in ORCHID‐BUD and ORCHID. Table S3. The number of cognitive domains tested for each patient. Table S4. Cognitive and psychological outcomes ‐ hydroxychloroquine vs placebo. Figure S1. Enrollment flow diagram. Figure S2. Potentially modifiable in‐hospital factors for 12‐month global cognition. Figure S3. Potentially modifiable in‐hospital factors for 12‐month executive function. Figure S4. Potentially modifiable in‐hospital factors for 12‐month immediate memory. Figure S5. Potentially modifiable in‐hospital factors for 12‐month verbal fluency. Figure S6. Potentially modifiable in‐hospital factors for 12‐month attention. Figure S7. Potentially modifiable in‐hospital factors for 12‐month abstraction. Figure S8. Potentially modifiable in‐hospital factors for 12‐month delayed memory. Figure S9. Potentially modifiable in‐hospital factors for 12‐month post‐traumatic stress disorder and depression. [file IRV-17-e13197-s001.docx]

**
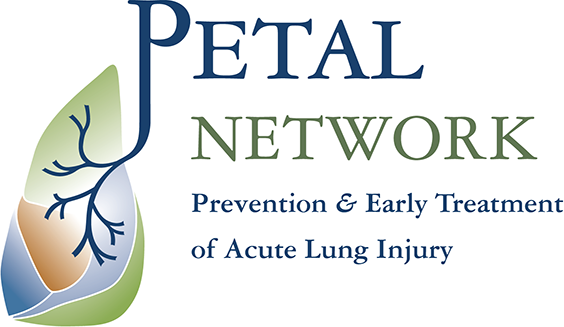
**

|  |  |
| --- | --- |

**ONLINE SUPPLEMENT**

**Modifiable In-hospital Risk Factors for 12-month Cognition, Post-traumatic Stress Disorder, and Depression in Adults Hospitalized with COVID-19**

| **Network:** | The Prevention and Early Treatment of Acute Lung Injury (PETAL) Clinical Trials Network |
| --- | --- |
| **Trial Title:** | Outcomes Related to COVID-19 treated with Hydroxychloroquine among In-patients with symptomatic Disease - Brain Outcomes and Psychological Distress (ORCHID-BUD) |
| **Funder:** | National Institute on Aging  National Heart, Lung, and Blood Institute (NHLBI) |
| **Corresponding Author:** | Jin H. Han, MD, MSc,  Critical Illness, Brain Dysfunction, and Survivorship Center, Vanderbilt University Medical Center  Geriatric Research, Education, and Clinical Center  Tennessee Valley Healthcare System  e-mail: [jin.h.han@vumc.org](mailto:jin.h.han@vumc.org) |

**Table of Contents**

**Appendices**

**Appendix A.** NHLBI PETAL Network Collaborators 3

**Appendix B.** Critical Illness, Brain Dysfunction, and Survivorship (CIBS) Center Collaborators 5

**Supplemental Tables**

**Supplemental Table 1.** Patient characteristics of those who were and were not enrolled 6

**Supplemental Table 2.** Patient characteristics of those who were enrolled in ORCHID-BUD and

ORCHID 7

**Supplemental Table 3.** The number of cognitive domains tested for each patient 8

**Supplemental Table 4.** Cognitive and psychological outcomes - hydroxychloroquine vs placebo 9

**Supplemental Figures**

**Supplemental Figure 1.** Enrollment flow diagram 10

**Supplemental Figure 2.** Potentially modifiable in-hospital factors for 12-month global cognition 11

**Supplemental Figure 3.** Potentially modifiable in-hospital factors for 12-month executive function 12

**Supplemental Figure 4.** Potentially modifiable in-hospital factors for 12-month immediate memory 13

**Supplemental Figure 5.** Potentially modifiable in-hospital factors for 12-month verbal fluency 14

**Supplemental Figure 6.** Potentially modifiable in-hospital factors for 12-month attention 15

**Supplemental Figure 7.** Potentially modifiable in-hospital factors for 12-month abstraction 16

**Supplemental Figure 8.** Potentially modifiable in-hospital factors for 12-month delayed memory 17

**Supplemental Figure 9.** Potentially modifiable in-hospital factors for 12-month post-traumatic

stress disorder and depression 18

**The National Heart, Lung and Blood Institute (NHLBI) Prevention and Early Treatment of Acute Lung Injury (PETAL) Clinical Trials Network:**

**Clinical Center or Clinical Coordinating Center Principal Investigator*

**ALIGNE Clinical Center**: Baystate Medical Center –Jay S Steingrub*, Howard Smithline, Bogdan Tiru, Mark Tidswell, Lori Kozikowski, Sherell Thornton-Thompson, Leslie De Souza; Brigham and Women’s Hospital –Peter C Hou*, Rebecca M Baron, Anthony F Massaro, Imoigele P Aisiku, Lauren E Fredenburgh, Raghu R Seethala, Lily Johnsky; Maine Medical Center –Richard R Riker, David B Seder, Teresa L May, Michael R Baumann, Ashley Eldridge, Christine Lord

**BOSTON Clinical Center:** Beth Israel Deaconess Medical Center –Nathan I Shapiro*, Daniel Talmor*,Thomas O’Mara, Charlotte Kirk, Kelly Harrison, Lisa Kurt, Margaret Schermerhorn, Valerie Banner-Goodspeed, Katherine Boyle, Nicole Dubosh; Massachusetts General Hospital –Michael Filbin, Kathryn Hibbert, Blair Alden Parry, Kendall Lavin-Parsons, Natalie Pulido, Brendan Lilley, Carl Lodenstein, Justin Margolin, Kelsey Brait; University of Mississippi Medical Center – Alan Jones, James Galbraith, Rebekah Peacock, Utsav Nandi, Taylor Wachs

**CALIFORNIA Clinical Center:** UCSF San Francisco –Michael Matthay*, Kathleen Liu, Kirsten Kangelaris, Ralph Wang, Carolyn Calfee, Kimberly Yee; UCLA– Gregory W Hendey*, Steven Y Chang, George Lim, Nida Qadir, Andrea Tam, Rebecca Beutler; Stanford University Hospital –Joseph E Levitt, Jenny G Wilson, Angela J Rogers, Rosemary Vojnik, Jonasel Roque; UC Davis – Timothy E Albertson, James A Chenoweth, Jason Y Adams, Skyler J Pearson, Maya M Juarez; UCSF Fresno –Eyad Almasri, Mohamed Fayed, Alyssa Hughes, Shelly Hillard; University of Texas – Ryan Huebinger, Henry Wang, Elizabeth Vidales, Bela Patel

**COLORADO Clinical Center**: University of Colorado Hospital –Adit A Ginde*, Marc Moss*, Amiran Baduashvili, Jeffrey McKeehan, Lani Finck, Carrie Higgins, Michelle Howell; Denver Health Medical Center –Ivor Douglas, Jason Haukoos, Terra Hiller, Carolynn Lyle, Alicia Cupelo, Emily Caruso, Claudia Camacho, Stephanie Gravitz; National Jewish Health Saint Joseph’s Hospital–James Finigan, Christine Griesmer

**MICHIGAN Clinical Center**: University of Michigan Medical Center –Pauline K Park*, Robert Hyzy*, Kristine Nelson, Kelli McDonough, Norman Olbrich; Indiana University – Mark Williams, Raj Kapoor, Jean Nash, Meghan Willig; Henry Ford – Jayna Gardner-Gray, Mayur Ramesh.

**MONTEFIORE-SINAI Clinical Center:** Montefiore Moses—Michelle Ng Gong*, Michael Aboodi, Ayesha Asghar, Omowunmi Amosu, Madeline Torres, Savneet Kaur; Montefiore Weiler –Jen-Ting Chen, Aluko Hope, Brenda Lopez, Kathleen Rosales, Jee Young You; University of Arizona— Jarrod M Mosier, Cameron Hypes, Bhupinder Natt, Bryan A Borg, Elizabeth Salvagio Campbell

**OHIO Clinical Center:**  University of Cincinnati Medical Center – R. Duncan Hite*, Kristin Hudock, Autumn Cresie, Faysal Alhasan, Jose Gomez-Arroyo Cleveland Clinic Foundation –Abhijit Duggal, Omar Mehkri, Andrei Hastings, Debasis Sahoo, Francois Abi Fadel, Susan Gole, Valerie Shaner, Allison Wimer, Yvonne Meli, Alexander King; Ohio State University Wexner Medical Center–Thomas E Terndrup*, Matthew C Exline, Sonal Pannu, Emily Robart, Sarah C Karow

**PACIFIC NORTHWEST Clinical Center:** Harborview Medical Center –Catherine L Hough*, Bryce RH Robinson*, Nicholas J Johnson, Daniel J Henning, Monica Campo, Stephanie Gundel, Sakshi Seghal, Sarah Katsandres, Sarah Dean; Oregon Health and Science University –Akram Khan, Olivia Krol, Milad Karami Jouzestani, Peter Huynh

**PITTSBURGH Clinical Center:** UPMC Presbyterian, UPMC Shadyside & UPMC Mercy—Alexandra Weissman, Donald M Yealy*, Denise Scholl, Peter W Adams, Bryan J McVerry, David Huang, Derek C Angus*, Penn State Hershey Medical Center – Jordan Schooler, Steven C Moore

**SOUTHEAST Clinical Center:** Wake Forest Baptist Health – D. Clark Files*, Chadwick Miller*, Kevin W Gibbs, Mary LaRose, Lori Flores, Lauren Koehler, Caryn G Morse, John Sanders, Caitlyn Langford, Kristen Nanney, Masiku Mdala-Gausi, Phyllis Yeboah; U. Kentucky–Peter E Morris, Jamie Sturgill, Sherif Seif, Evan P Cassity, Sanjay Dhar; Virginia Commonwealth University Medical Center–Marjolein de Wit, Jessica Mason; Medical University of South Carolina – Andrew J Goodwin, Greg Hall, Abbey Grady, Amy Chamberlain

**UTAH:** Intermountain Medical Center – Samuel M Brown*, Joseph Bledsoe*, Lindsay M Leither, Ithan Peltan, Nathan Starr, Melissa Fergus, Valerie Aston, Quinn Montgomery, Rilee Smith, Mardee Merrill, Katie Brown, Brent Armbruster; University of Utah Hospital –Estelle Harris, Elizabeth A Middleton, Robert Paine III, Stacy A Johnson, Macy Barrios

**VANDERBILT Clinical Center:** Duke University Medical Center—John Eppensteiner, Alexander T Limkakeng, Lauren McGowan, Tedra Porter, Andrew Bouffler, J. Clancy Leahy; Louisiana State University Health Sciences Center—Bennet deBoisblanc, Matthew Lammi, Kyle Happel, Paula Lauto; Vanderbilt University Medical Center –Wesley H Self*, Jonathan D Casey, Matthew W Semler, Sean P Collins, Frank Harrell, Christopher J Lindsell, Todd W Rice*, William B Stubblefield, Christopher S Gray, Jakea Johnson, Megan Roth, Margaret Hays, Donna Torr, Arwa Zakaria

**Clinical Coordinating Center:** Massachusetts General Hospital Biostatistics Center: David A Schoenfeld*, B. Taylor Thompson*, Douglas L Hayden, Nancy Ringwood, Cathryn Oldmixon, Christine Ulysse, Richard Morse, Ariela Muzikansky, Laura Fitzgerald, Samuel Whitaker, Adrian Lagakos

**Steering Committee Chair:** Roy G Brower

**National Heart, Lung, and Blood Institute**: Lora Reineck, Neil Aggarwal, Karen Bienstock, Michelle Freemer, Myron Maclawiw, Gail Weinmann

**Appendix B. Critical Illness, Brain Dysfunction, and Survivorship (CIBS) Center**

**Clinical Core:** Karen Sheppard (Study Coordinator), Chris Berkey

**Long-term Outcomes Core:** Alana Lauck (Study Coordinator), Aidan Wells, Cade Bennett, Paul Allen, Jordan Clemens, Katie Bowder

**Supplemental Table 1.** Patient characteristics stratified by patients who completed the 12-month follow up, were without contact information, were lost to follow-up, refused to participate, and were non-English or non-Spanish speaking.

| **Variable** | **Completed 12-month follow-up n=203** | **No contact information**  **n=20** | **Lost to follow-up**  **n=91** | **Refused**  **n=43** | **Non-English / non-Spanish speaking**  **n=21** |
| --- | --- | --- | --- | --- | --- |
| Median (IQR) age, years | 55.0 (43.5, 63.0) | 47.5 (40.2, 68.5) | 48.0 (40.5, 60.5) | 61 (52, 67.0) | 65.0 (51.0, 79.0) |
| Female Sex | 94 (46.3%) | 10 (50.0%) | 30 (33.0%) | 19 (44.2%) | 10 (47.6%) |
| Race/Ethnicity Combined |  |  |  |  |  |
| Hispanic/Latinx | 84 (41.4%) | 7 (35.0%) | 60 (65.9%) | 7 (16.3%) | 0 (0.0%) |
| Non-Hispanic White | 63 (31.0%) | 8 (40.0%) | 6 (6.6%) | 12 (27.9%) | 4 (19.0% |
| Non-Hispanic Black | 47 (23.2%) | 4 (20.0%) | 15 (16.5%) | 15 (34.9%) | 7 (33.3%) |
| Non-Hispanic Asian | 3 (1.5%) | 0 (0.0%) | 0 (0.0%) | 2 (4.7%) | 4 (19.0%) |
| Non-Hispanic American Indian or Alaskan Native | 3 (1.5%) | 1 (5.0%) | 3 (3.3%) | 2 (4.7%) | 1 (4.8%) |
| Non-Hispanic Native Hawaiian or Other Pacific Islander | 1 (0.5%) | 0 (0.0%) | 1 (1.1%) | 2 (4.7%) | 0 (0.0%) |
| Non-Hispanic Multiple Race | 2 (1.0%) | 0 (0.0%) | 1 (1.1%) | 0 (0.0%) | 1 (4.8%) |
| Non-Hispanic Other Race | 0 (0.0%) | 0 (0.0%) | 3 (3.3%) | 1 (2.3%) | 3 (14.3%) |
| Unknown Ethnicity | 0 (0.0%) | 0 (0.0%) | 2 (2.2%) | 2 (4.7%) | 1 (4.8%) |
| Homeless or living only in temporary residences before hospitalization | 1 (0.5%) | 3 (17.6%) | 1 (1.1%) | 1 (2.3%) | 0 (0.0%) |
| Median (IQR) BMI, kg/m^2^ | 32.0 (27.9, 37.2) | 33.5 26.7,39.5) | 31.2 (27.2, 37.2) | 31.8 (27.7 38.3) | 27.2 (24.6, 31.2) |
| Past medical history |  |  |  |  |  |
| Hypertension | 99 (48.8%) | 10 (50.0%) | 38 (41.8%) | 21 (48.8%) | 13 (61.9%) |
| Diabetes Mellitus | 63 (31.0%) | 8 (40.0%) | 32 (35.2%) | 14 (32.6%) | 7 (33.3%) |
| Coronary artery disease | 9 (4.4%) | 1 (5.0%) | 7 (7.7%) | 2 (4.7%) | 2 (9.5%) |
| Chronic obstructive pulmonary disease | 13 (6.4%) | 1 (5.0%) | 4 (4.4%) | 3 (7.0%) | 1 (4.8%) |
| Chronic kidney disease | 16 (7.9%) | 2 (10.0%) | 7 (7.7%) | 2 (4.7%) | 2 (9.5%) |
| SOFA score at enrollment | 2.00 (1.00, 3.00) | 2.50 (1.00, 3.25) | 2.00 (1.00,4.00) | 2.00 (1.00, 3.00) | 2.00 (1.00, 3.00) |
| Ever in the ICU | 63 (31.0%) | 6 (30.0%) | 30 (33.0%) | 11 (25.6%) | 3 (14.3%) |
| Median (IQR) ICU length of stay for all patients, days* | 0.00 (0.00, 3.50) | 0.00 (0.00, 3.25) | 0.00 (0.00, 2.50) | 0.00 (0.00, 0.00) | 0.00 (0.00, 0.00) |
| Ever mechanically ventilated | 34 (16.7%) | 3 (15.0%) | 11 (12.1%) | 4 (9.3%) | 2 (9.5%) |
| Ever vasopressor use | 27 (13.3%) | 3 (15.0%) | 12 (13.2%) | 5 (11.6%) | 2 (9.5%) |

IQR, interquartile range; SOFA, Sequential Organ Failure Assessment.

#Pre-existing dementia determined by a past history of dementia, home cholinesterase inhibitor use, or an IQCODE > 3.3.

* Length of stay and duration calculated from randomization to 28 days.

| **Variable** | **Completed 12-month follow-up**  **n=203** | **ORCHID Hydroxychloroquine**  **n=242** | **ORCHID**  **Placebo**  **n=237** |
| --- | --- | --- | --- |
| Median (IQR) age, years | 55.0 (43.5, 63.0) | 58 (45, 69) | 57 (43, 68) |
| Female Sex | 94 (46.3%) | 107 (44.2%) | 105 (44.3%) |
| Race/Ethnicity Combined |  |  |  |
| Hispanic/Latinx | 84 (41.4%) | 91 (39.2%) | 87 (38.3%) |
| Non-Hispanic White | 63 (31.0%) | 72 (31.0%) | 65 (28.6%W) |
| Non-Hispanic Black | 47 (23.2%) | 57 (24.6%) | 55 (24.2%) |
| Non-Hispanic Asian | 3 (1.5%) | 4 (1.7%) | 7 (3.1%) |
| Non-Hispanic American Indian or Alaskan Native | 3 (1.5%) | 5 (2.2%) | 8 (3.5%) |
| Non-Hispanic Native Hawaiian or Other Pacific Islander | 1 (0.5%) | 2 (0.9%) | 4 (1.8%) |
| Non-Hispanic Multiple Race | 2 (1.0%) | 1 (0.4%) | 1 (0.4%) |
| Non-Hispanic Other Race | 0 (0.0%) | 0 (0.0%) | 0 (0.0%) |
| Unknown Ethnicity | 0 (0.0%) | 0 (0.0%) | 0 (0.0%) |
| Homeless or living only in temporary residences before hospitalization | 1 (0.5%) | - | - |
| Median (IQR) BMI, kg/m^2^ | 32.0 (27.9, 37.2) | 31.3 (26.4, 37.2) | 31.1 (27.2, 36.5) |
| Past medical history |  |  |  |
| Hypertension | 99 (48.8%) | 136 (56.2%) | 117 (49.4%) |
| Diabetes Mellitus | 63 (31.0%) | 88 (36.4%) | 78 (32.9%) |
| Coronary artery disease | 9 (4.4%) | 19 (7.9%) | 23 (9.7%) |
| Chronic obstructive pulmonary disease | 13 (6.4%) | 18 (7.4%) | 21 (8.9%) |
| Chronic kidney disease | 16 (7.9%) | 28 (11.6%) | 14 (5.9%) |
| SOFA score at enrollment | 2.00 (1.00, 3.00) | 2 (1, 4) | 2 (1, 4) |
| Ever in the ICU | 63 (31.0%) | - | - |
| Median (IQR) ICU length of stay for all patients, days* | 0.00 (0.00, 3.50) | - | - |
| Ever mechanically ventilated | 34 (16.7%) | 41 (16.9%) | 47 (19.8%) |
| Ever vasopressor use | 27 (13.3%) | - | - |
| IQR, interquartile range; SOFA, Sequential Organ Failure Assessment.  #Pre-existing dementia determined by a past history of dementia, home cholinesterase inhibitor use, or an IQCODE > 3.3.  - Not reported in the original ORCHID paper.  * Length of stay and duration calculated from randomization to 28 days. | | | |

**Supplemental Table 2.** Patient characteristics stratified by patients who completed the 12-month follow up and were enrolled in the Outcomes Related to COVID-19 Treated With Hydroxychloroquine Among Inpatients With Symptomatic Disease (ORCHID) parent trial

**Supplemental Table 3.** The number of cognitive domains completed by the 201 patients who participated in the neuropsychological battery. These cognitive domains contributed to the Cognitive Composite Score.

| **The Number of Cognitive Domains Completed by Patients** | **n (%)** |
| --- | --- |
| 6 | 189 (93.1%) |
| 5 | 4 (2.0%) |
| 4 | 2 (1.0%) |
| 3 | 2 (1.0%) |
| 2 | 1 (0.5%) |
| 1 | 3 (1.5%) |

| **Outcomes at 12-months** | **HCQ**  **Total n=97** | **N for HCQ** | **Placebo**  **Total n=106** | **N for placebo** |
| --- | --- | --- | --- | --- |
| **Cognitive Outcomes** |  |  |  |  |
| Cognitive Composite score, median (IQR) | -1.10 (-1.74, -0.54) | 97 | -1.03 (-1.76, -0.55) | 104 |
| MOCA-Blind score, median (IQR) | 16.0 (13.0, 18.0) | 97 | 16.0 (13.0, 18.0) | 105 |
| Individual Cognitive Domain Z-scores |  |  |  |  |
| Executive function - Hayling Sentence Completion, median (IQR) | -1.94 (-3.19, -0.06) | 95 | -1.31 (-2.56, -0.69) | 103 |
| Immediate memory - Craft Story, median (IQR) | -1.55 (-2.04, -0.57) | 97 | -1.15 (-2.28, -0.49) | 101 |
| Attention – WAIS-IV Digit Span, median (IQR) | -1.33 (-0.67, 0.33) | 95 | -1.33 (-0.67, 0.33) | 102 |
| Verbal fluency - COWA, median (IQR) | -1.00 (-1.60, 0.10) | 93 | -0.90 (-1.60, -0.13) | 102 |
| Abstraction – WAIS-IV Similarities, median (IQR) | -0.67 (-1.67, 0.00) | 93 | -0.67 (-1.33, 0.00) | 101 |
| Delayed memory – Craft Story, median (IQR) | -1.66 (-2.38, -0.79) | 93 | -1.34 (-2.28, -0.47) | 98 |
|  |  |  |  |  |
| **Psychological Outcomes** |  |  |  |  |
| PCL-5 Score, median (IQR) | 11.0 (4.0, 19.8) | 92 | 10.0 (3.0, 23.0) | 102 |
| HADS-Depression Score, median (IQR) | 5.0 (2.0, 9.0) | 92 | 4.0 (1.0, 8.0) | 102 |

**Supplemental Table 4.** Cognitive and psychological outcomes in hospitalized COVID-19 patients who received hydroxychloroquine (HCQ) versus placebo.

Global cognition was characterized a Cognitive Composite Score which took the average of all the cognitive domain Z-scores and the MOCA-Blind. Lower scores represent lower cognitive performance. Post-traumatic stress disorder (PTSD) was assessed for using the PTSD Checklist for the DSM-V (PCL-5) and depression was assessed for using the Hospital Anxiety Depression Scale (HADS) Depression Subscale, respectively. Higher scores represented more severe symptoms.

HCQ, hydroxychloroquine; IQR, interquartile range; WAIS-IV, Wechsler Adult Intelligence Scale-IV; COWA, Controlled Oral Word Association Test; HADS, Hospital Anxiety and Depression Questionnaire.


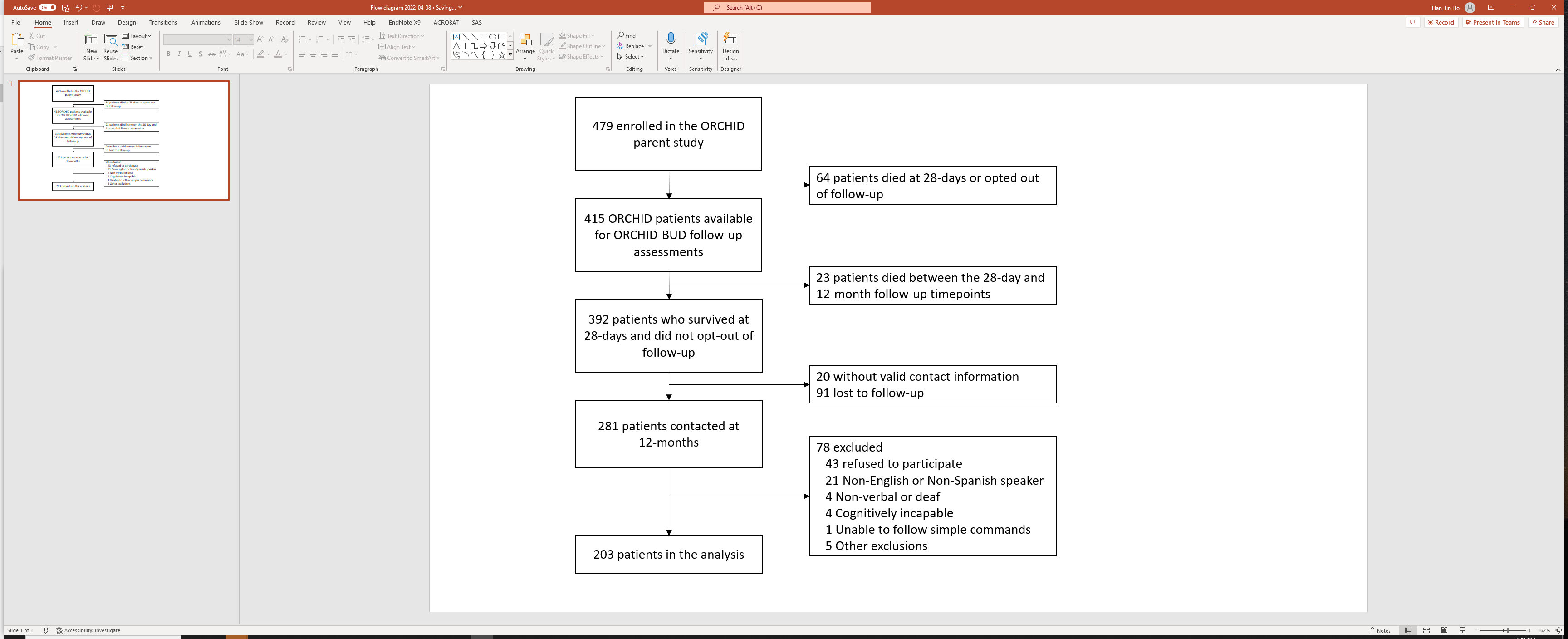


**Supplemental Figure 1.** Enrollment flow diagram.


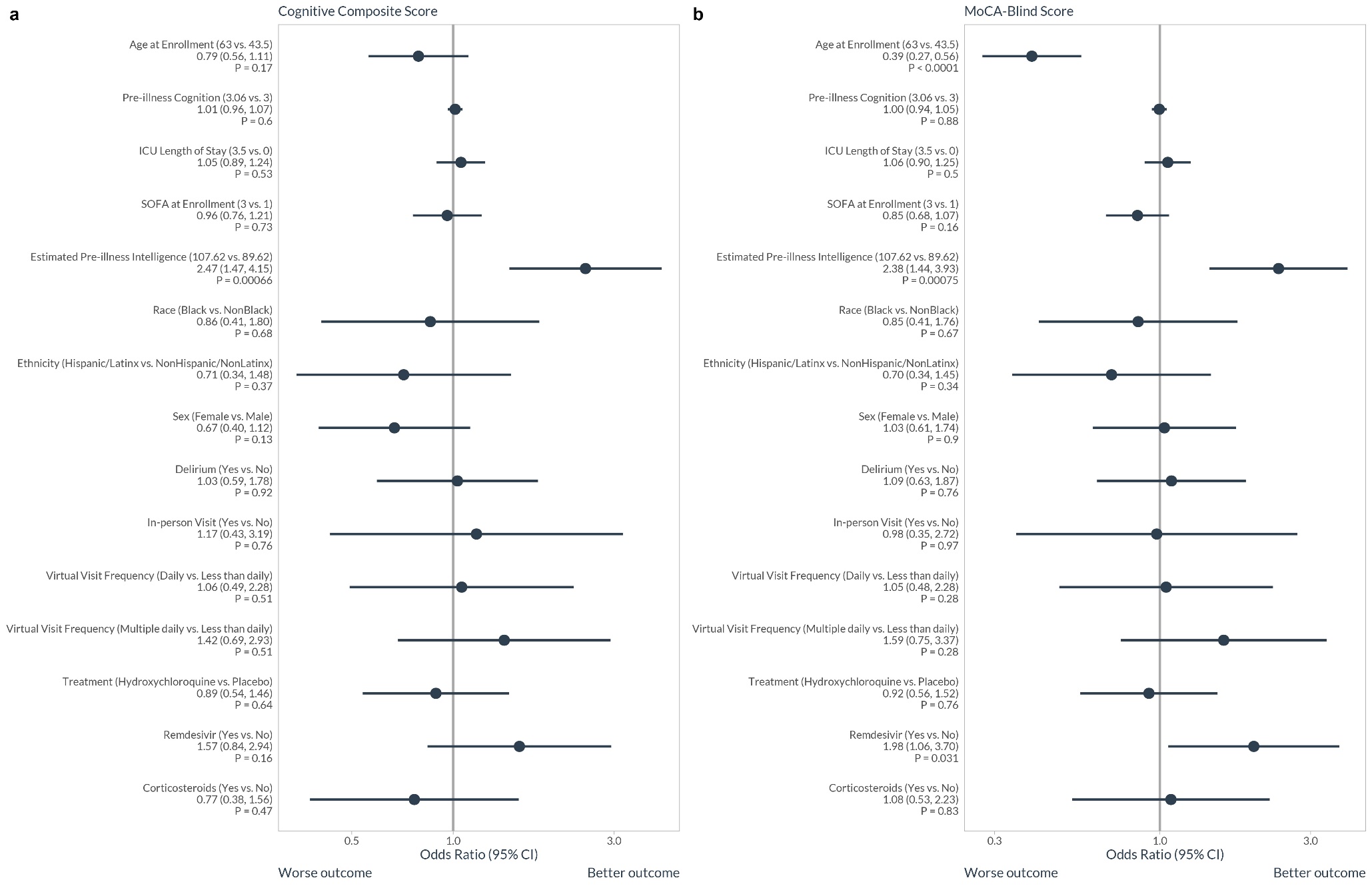


**Supplemental Figure 2.** The full proportion odds logistic regression model evaluating potentially modifiable in-hospital factors during the treatment of acute COVID-19 on 12-month global cognition as characterized by the (a) Cognitive Composite Score based on a detailed neuropsychological battery and (b) MOCA-Blind. Pre-illness cognition was characterized by the short form Informant Questionnaire on Cognitive Decline in the. Elderly. Intelligence was estimated using the Barona Index.

ICU, intensive care unit; SOFA, Sequential Organ Failure Assessment.


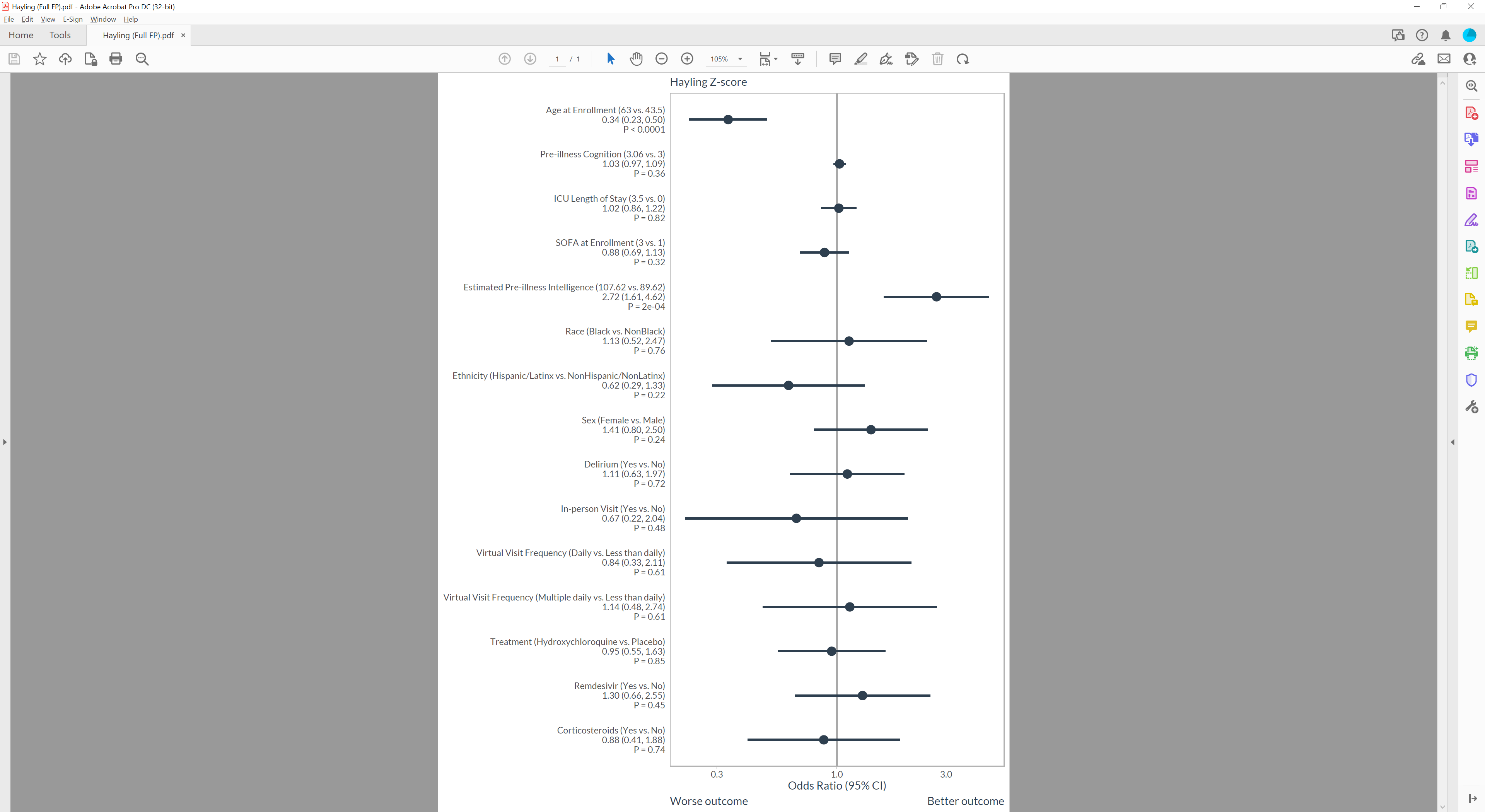


**Supplemental Figure 3.** The full proportion odds logistic regression model evaluating potentially modifiable in-hospital factors during the treatment of acute COVID-19 on 12-month executive function as characterized by the Hayling Sentence Completion Test Z-score.


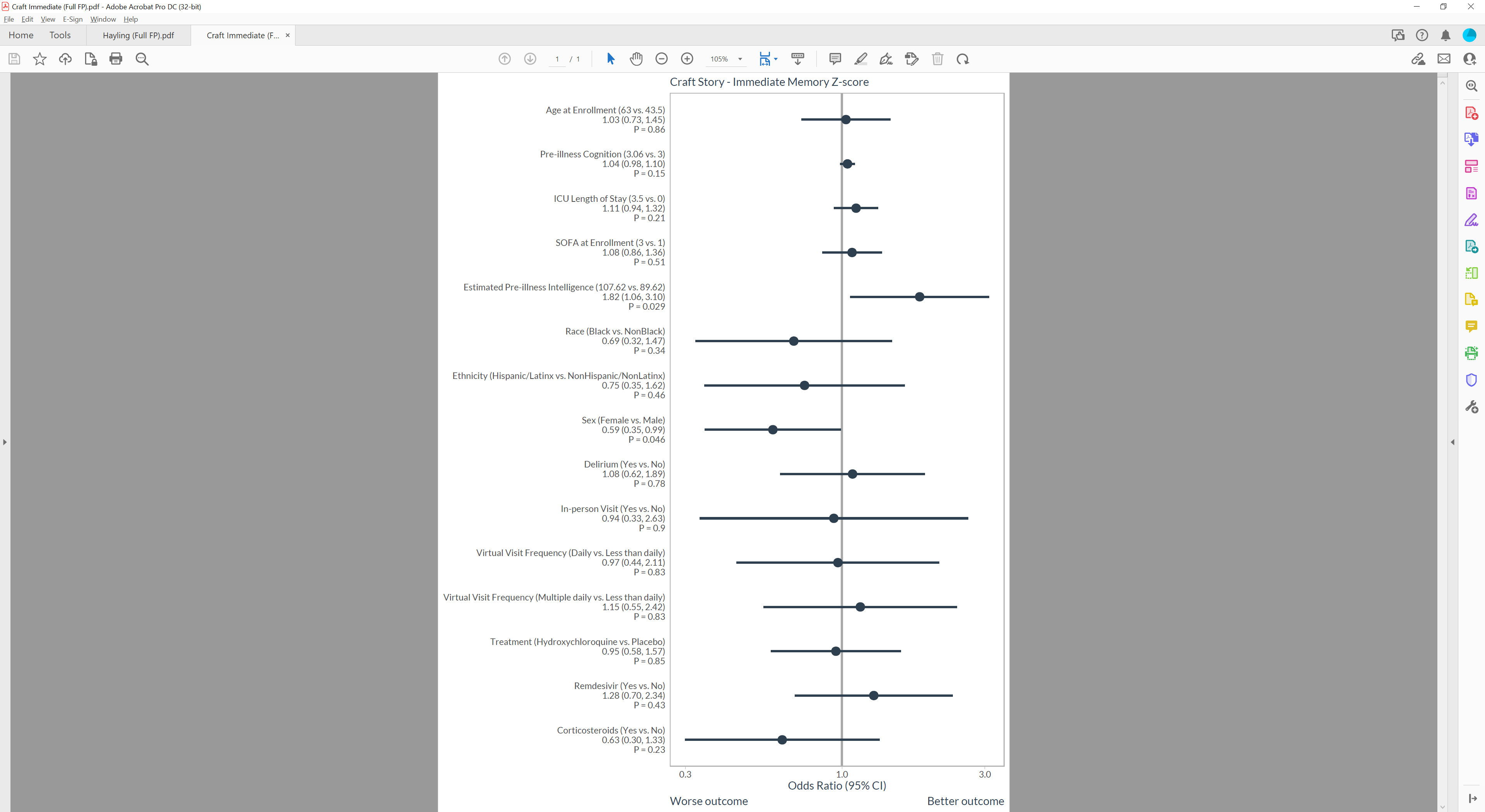


**Supplemental Figure 4.** The full proportion odds logistic regression model evaluating potentially modifiable in-hospital factors during the treatment of acute COVID-19 on 12-month immediate memory as characterized by the Craft Story – Immediate Memory Test Z-score.


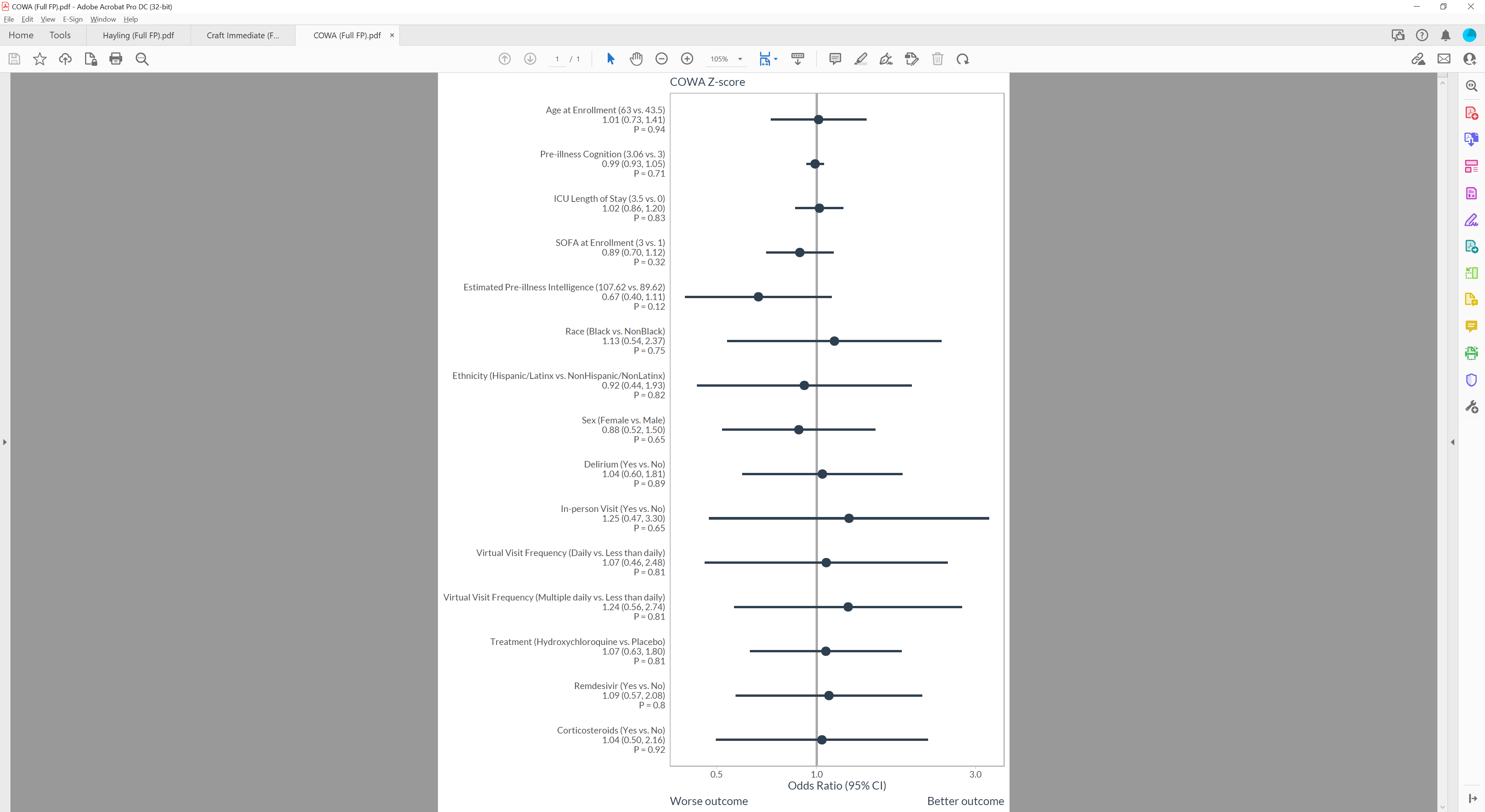


**Supplemental Figure 5** The full proportion odds logistic regression model evaluating potentially modifiable in-hospital factors during the treatment of acute COVID-19 on 12-month verbal fluency as characterized by the characterized by the Controlled Oral Word Association (COWA) test Z-score.


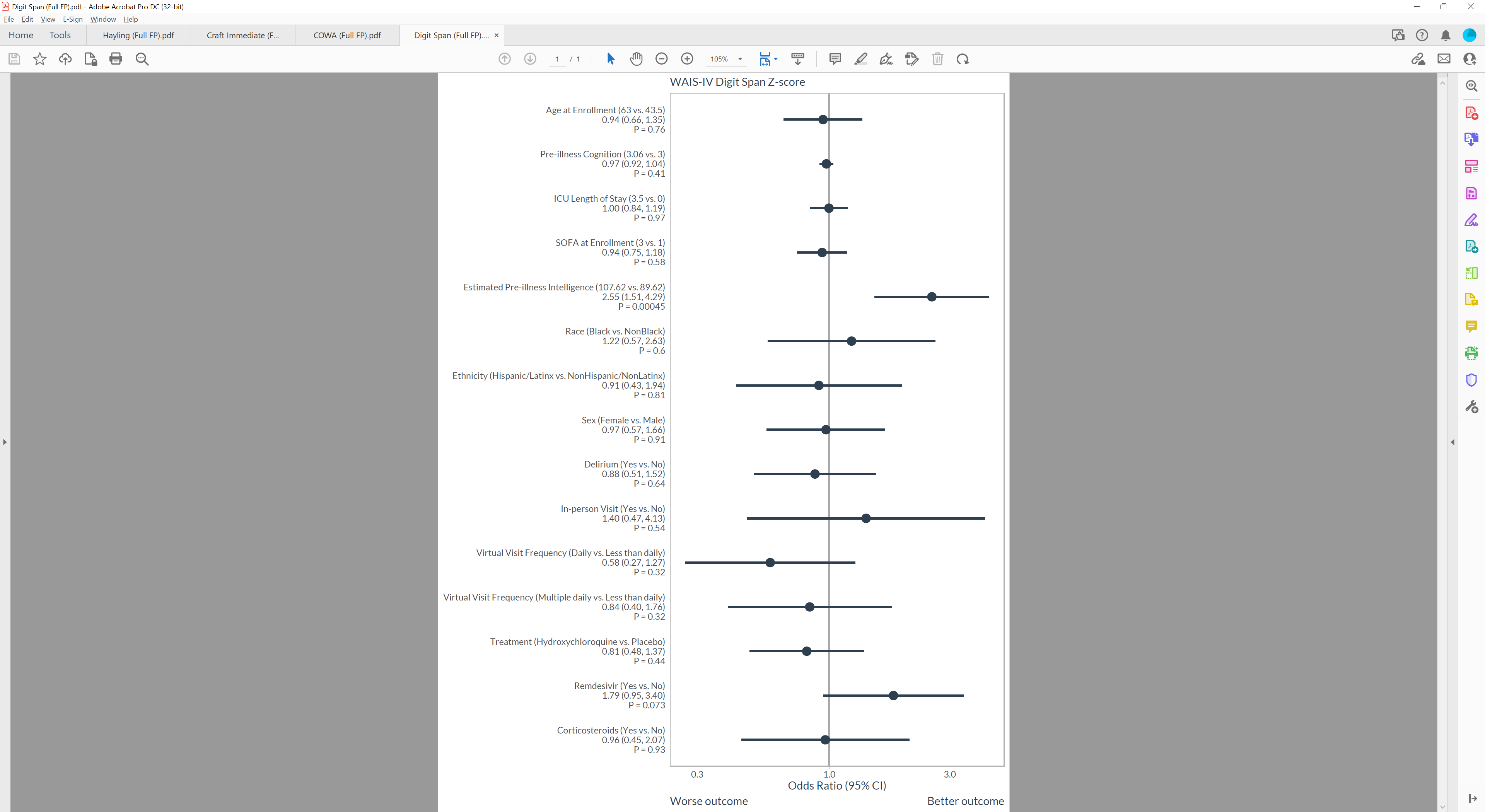
**Supplemental Figure 6.** The full proportion odds logistic regression model evaluating potentially modifiable in-hospital factors during the treatment of acute COVID-19 on 12-month attention as characterized by the Wechsler Adult Intelligence Scale-IV (WAIS-IV) Digit Span test Z-score.


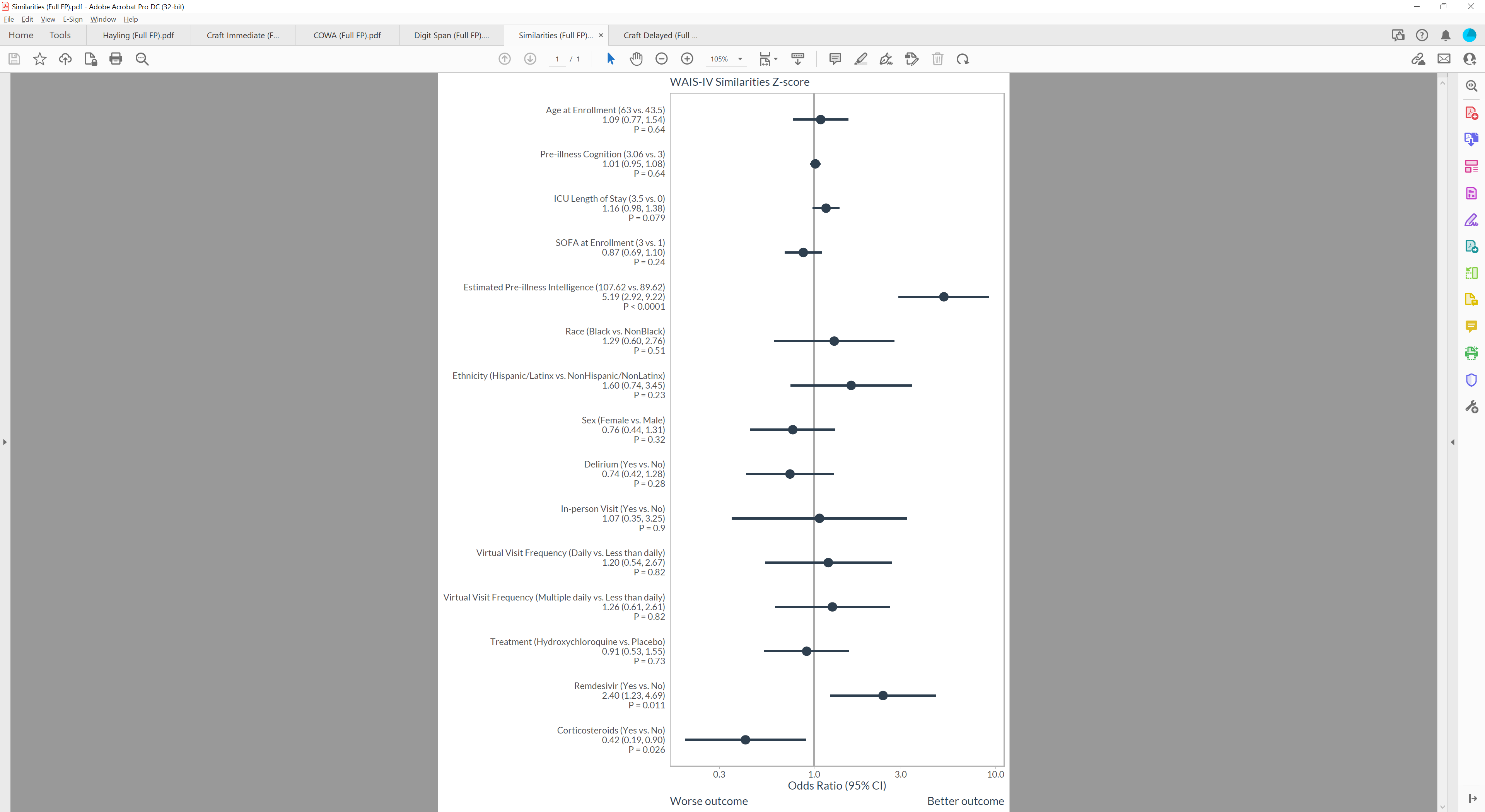
**Supplemental Figure 7.** The full proportion odds logistic regression model evaluating potentially modifiable in-hospital factors during the treatment of acute COVID-19 on 12-month abstraction as characterized by the Wechsler Adult Intelligence Scale-IV (WAIS-IV) Similarities test Z-score.


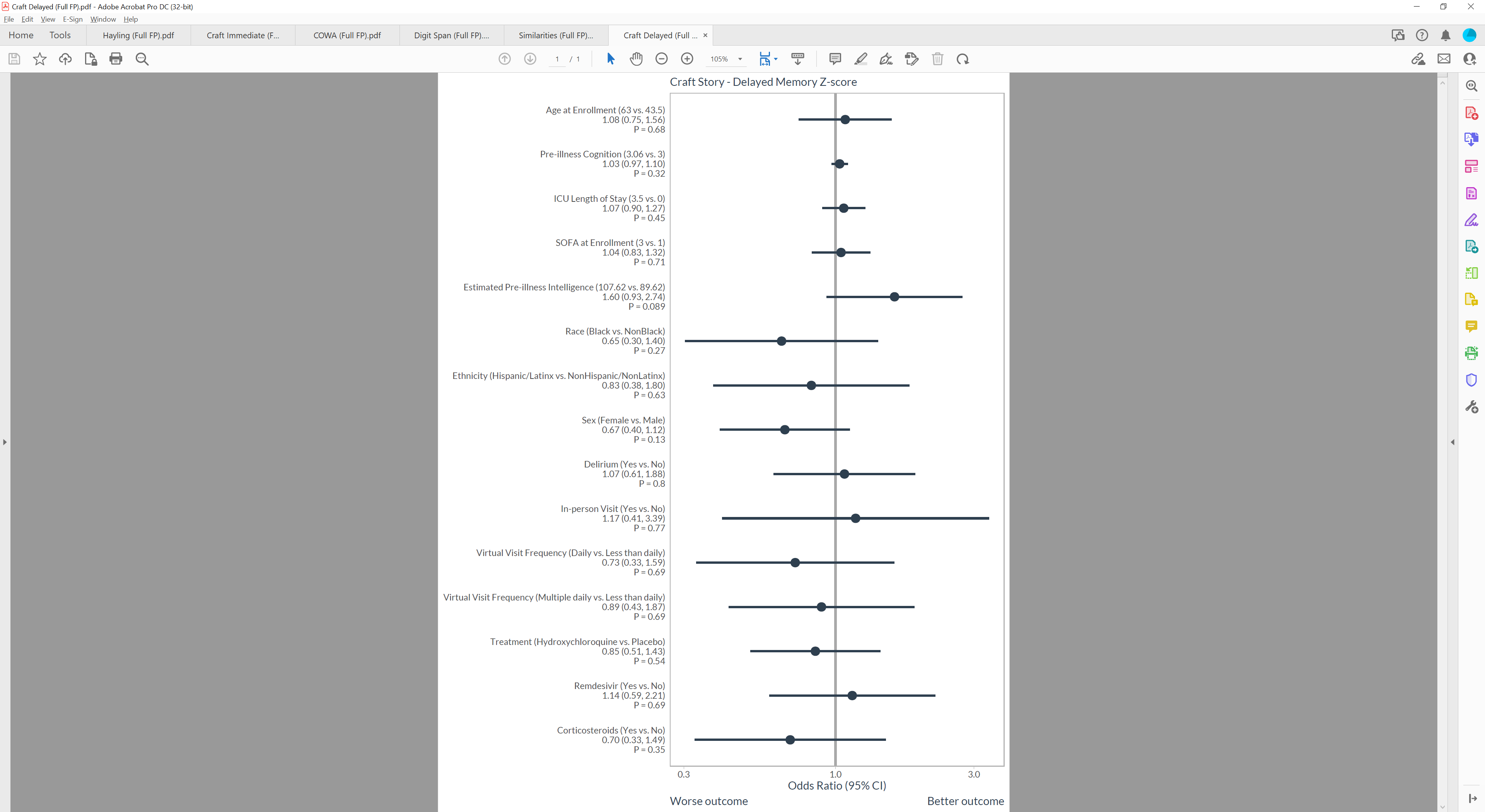
**Supplemental Figure 8.** The full proportion odds logistic regression model evaluating potentially modifiable in-hospital factors during the treatment of acute COVID-19 on 12-month delayed memory as characterized by the Craft Story – Delayed Memory test Z-score.

**
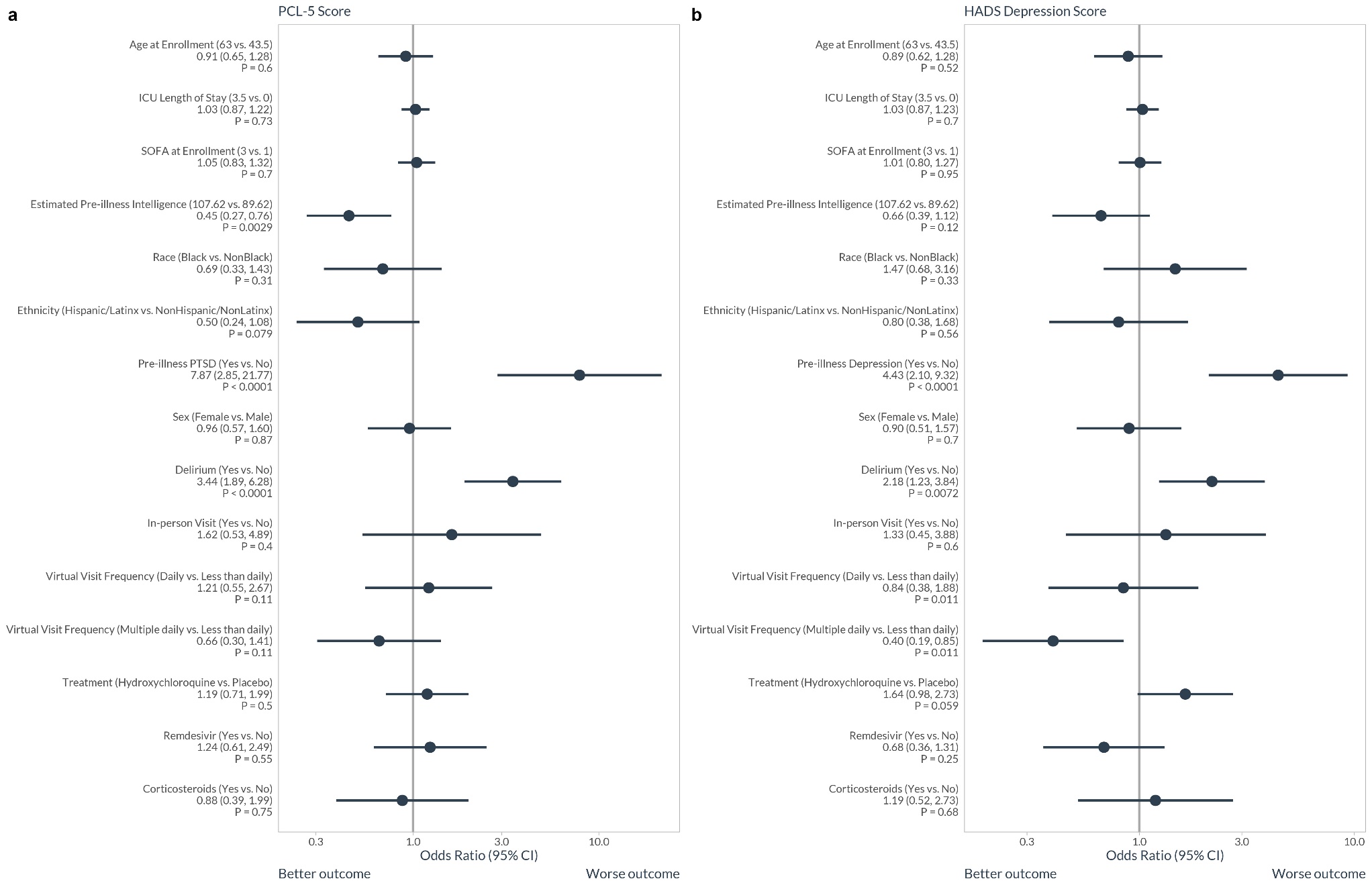
 Supplemental Figure 9.** The full proportion odds logistic regression model evaluating potentially modifiable in-hospital factors during the treatment of acute COVID-19 on 12-month (a) post-traumatic stress disorder (PTSD) symptoms as characterized by the PTSD Checklist for the DSM-V (PCL-5) and (b) depression symptoms as characterized by Hospital Anxiety Depression Scale (HADS) – Depression subscale. Intelligence was estimated using the Barona Index. ICU, intensive care unit; SOFA, Sequential Organ Failure Assessment. Pre-illness PTSD and depression as obtained by patient and family interview.
